# Reducing Adverse Drug Reactions for Older People in the Community: Evaluating the Validity and Reliability of the ADRe Profile

**DOI:** 10.1155/jonm/9921349

**Published:** 2025-05-14

**Authors:** Vera Logan, Neil Carter, David Hughes, Adam Turner, Sue Jordan

**Affiliations:** ^1^Faculty of Health and Social Sciences, University of South Bohemia, Ceske Budejovice, Czech Republic; ^2^Faculty of Medicine, Health and Life Science, Swansea University, Swansea, UK

**Keywords:** drug-related side effects and adverse reactions, pharmacovigilance, primary health care, validation study

## Abstract

**Background:** Adverse drug reactions (ADRs), particularly in the context of polypharmacy, remain a persistent, unresolved problem for patients and healthcare professionals. The ADRe Profile identifies medicine-related harms, and supports their resolution, thereby improving care quality and preventing future problems.

**Objective:** The objective of this study was to assess the validity and reliability of the ADRe Profile (https://www.swansea.ac.uk/adre/) in U.K. primary care general practices, building on assessments in other settings.

**Methods:** The ADRe Profile's validity and reliability were investigated using complementary mixed methods: content validity index, contrast group construct validity, cognitive interviewing, and inter-rater reliability.

**Results:** Cognitive interviews (*n* = 5) confirmed that the ADRe Profile needed only minor adjustments. The scale-level content validity index was 0.67 (*n* = 14), items ranging from 0.08 to 1. Significant differences in signs and symptoms associated with ADRs between service users taking different numbers of regular prescribed medicines confirmed construct validity (*n* = 68, *U* = 870.50, *p* < 0.001). Inter-rater reliability testing showed substantial agreement between service users and research nurse: 10 items had 100% agreement. Overall *kappa* mean was 0.71 (range: 0.31–1), (*n* = 42).

**Conclusions and Relevance:** The ADRe Profile is suitable for use with older service users in primary care who live at home. Users understood the questions and provided meaningful answers. ADRe Profile responses were sufficiently reliable to be used as a basis for further investigations, prescriber referral and clinical actions. However, clinician judgement of content validity may depend on knowledge and experience, highlighting the importance of training. Clinicians acknowledged that the ADRe Profile was comprehensive but identified practical difficulties. Instruments to reduce ADRs should be validated before testing in feasibility studies and randomised controlled trials.

**Implications for Nursing Management:** Managers need to optimise patient safety by introducing patient-centred symptom monitoring, with decision support. Before instruments are adopted, managers should check the reliability and validity data.

**Trial Registration:** ClinicalTrials.gov identifier: NCT04663360

## 1. Introduction and Background

Adverse drug reactions (ADRs) are defined as ‘appreciably harmful or unpleasant reactions, resulting from an intervention related to the use of a medicinal product, which predicts hazard from future administration and warrants prevention or specific treatment, or alteration of the dosage regimen, or withdrawal of the product' [1], p. 1255. The ADRe Profile (https://www.swansea.ac.uk/adre/) is an interdisciplinary instrument based on an ADR checklist complemented by decision support, encompassing information on potential aetiology, additional assessments and strategies for monitoring or addressing problems at the click of an icon.

As more people are prescribed medicines for long-term conditions, the problem of ADRs is increasing [2, 3]. ADRs are pervasive: A systematic review and meta-analysis in primary care revealed an ADR prevalence of 28.43% among the elderly (> 65 years) [4]. In the United Kingdom, ADR-related unplanned hospital admissions count for 8.3% of the total [5]. ADR-related admissions and bed-days increased by over 50% between 2008 and 2015 [6]. Systematic reviews estimate ADRs affect one in 6 hospitalised patients [7], rising to 85% among elderly individuals with dementia [8]. ADRs attributable to error cause 712 deaths directly and contribute to another 1708 each year [9]. These statistics have generated interest in the monitoring and management of ADRs.

Current approaches to monitoring ADRs or side effects of medicines are unable to mitigate the harm emanating from ADRs [10, 11]. Medicine chart reviews [12, 13] and patient self-reports are the most common approaches to monitoring ADRs; although these improve prescribing, there is little impact on patient outcomes [14–16]. There is an urgent need to develop better ways of identifying and reducing ADRs to control this hidden epidemic [2]. However, to ensure that volunteers' time contributes to worthwhile developments, relevant instruments must be validated with their target population before implementation in clinical trials and practice [17].

Reliability and validity are pivotal to assessment of instruments that measure changes in health status or quality of life [18, 19]. It is difficult to claim clinical effectiveness for healthcare interventions if information on their reliability and validity is not in the public domain [20, 21]. The evaluation of measurement techniques allows a comprehensive understanding of the potential biases inherent in the utilization of specific instruments [22]. Absence of validity data risks meaningless and inaccurate outcomes [23]. Inferiorly executed randomised controlled trials (RCTs) are more likely to yield biased outcomes than well-conducted RCTs or nonrandomised studies [24]. Accordingly, the development of the ADRe Profile has followed a trajectory of iterative refinement and publications relating to reliability and validity [25–34]. Initially, the checklist items were assembled from evidence-based sources, mainly the British National Formulary and manufacturers' summaries of product characteristics (SmPCs), and curated by a multidisciplinary team comprising expert doctors, pharmacists, academics and nurses. Subsequent iterations involved ongoing adjustments to both the items and the accompanying supporting information, informed by implementation experience.

Before using the ADRe Profile in a RCT evaluating the clinical impact of its implementation in older polymedicated adults living in their own homes, validity, reliability and feasibility needed to be established in this group. study protocol is published [35].

This paper describes evaluation of the validity and reliability of the ADRe Profile. Feasibility testing, undertaken to ascertain whether the ADRe Profile could be implemented in practice, and the trial results will be reported in future publications.

## 2. Methods

### 2.1. The Study Design

**Figure 1:**
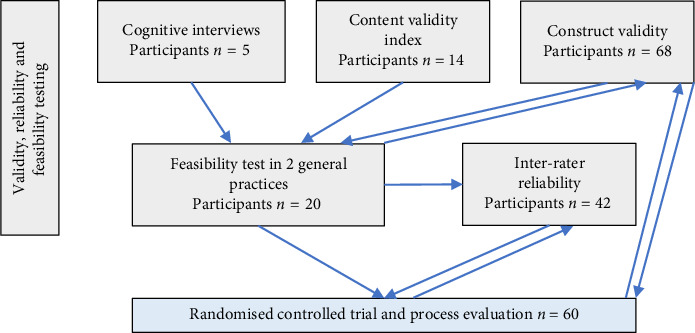
Flowchart of reliability and validity testing.

To evaluate the reliability and validity of the ADRe Profile, both qualitative evidence and quantitative evidence were gathered. Content validity was established through cognitive interviews and the calculation of a content validity index (CVI). Construct validity, which assesses whether the concept's application relates to the number of medicines taken, rather than other situational factors, was confirmed by implementing the ADRe Profile in contrast groups. Inter-rater reliability (IRR) was measured with Cohen's kappa coefficient using data from both the feasibility test and the intervention arm of the RCT. The evaluation of the ADRe Profile's validity and reliability was conducted in accordance with the definitions provided by the Consensus-based Standards for the Selection of Health Measurement Instruments (COSMIN) [36] and the Guidelines for Reporting Reliability and Agreement Studies (GRRAS) [37]. A flow chart detailing the sequence of tests conducted is provided (Figure 1).

### 2.2. Setting and Participants

Participants were recruited in Southwest Wales between April 2021 and July 2022, through advertising in the Wales Centre for Primary and Emergency Care Research (PRIME), research networks and Swansea University. Cognitive interviews were undertaken with service users aged 65 or over and healthcare professionals in primary care who might use the ADRe Profile. A CVI was then calculated. Construct validity testing involved two groups of 20 and 48 service users aged 65 or more: one group without any regular medicines and a second group prescribed five or more items daily. The latter were recruited from six general practices in Southwest Wales; these participants completed the ADRe Profile with one researcher (VL). IRR testing was calculated with 42 patients using 5 or more regular medicines from 5 practices (see Figure 1 for an outline). These participants were recruited from the feasibility study plus a further three general practices in Southwest Wales. (Further details are described in [35]).

### 2.3. Ethical Approval

Ethical approval was granted by the Health Research Authority Research Ethics Committee on 17 March 2021 (Integrated Research Application System [IRAS] 292693). All participants gave informed, signed consent to their participation.

### 2.4. Cognitive Interviews

To ensure the accuracy and functionality of the ADRe Profile, cognitive interviews were conducted following established procedures [38–40], in accordance with the methodological framework of the Cognitive Interviewing Reporting Framework of Boeije and Willis [41]. Think-aloud/read aloud techniques, augmented by concurrent probes [38, 42], were employed, and interpretations of the questions were noted.

Five participants were approached for cognitive interviews, and all agreed. The determination of sample size considered:

1. The nature of the questionnaire, whereby concrete questions about the respondent's health and well-being may necessitate fewer cognitive interviews than exploration of more abstract issues [43].
2. Prior cognitive interview testing of earlier versions of the ADRe Profile, with almost the same content [31, 32].

The sample was matched to the intended target population of users and recipients of the ADRe Profile. Two patients aged > 64, a nurse, a pharmacist and a general practitioner (GP) participated. Interviews were conducted by a single researcher (VL) via the Zoom video conferencing platform at mutually convenient times between April and October 2021. Face-to-face interviews were not possible due to the pandemic.

The interview protocol underwent refinement following a preliminary ‘test run' within the research team [44]. An audio recording of each interview was made to facilitate thorough analysis, while the interviewer maintained detailed notes to capture immediate insights. Since Willis [45] suggests that cognitive interviews are not typically transcribed, we relied on detailed notes and recordings to capture participants' responses.

Comments pertaining to specific questionnaire items were coded into predefined categories, informed by the Question Appraisal System (QAS-99) [46]; any responses not aligning with the predetermined categories were labelled as ‘other', and thematic reduction techniques were applied to identify overarching themes. Data reduction techniques [47] were employed to condense and organise the data into meaningful categories.

### 2.5. CVI

To ascertain whether the items on the ADRe Profile represent health and well-being problems associated with multiple medicines commonly prescribed for older individuals receiving primary care, a CVI was calculated, following established methods [48–50]. From professional contacts within the Faculty of Medicine, Health and Life Science, a patient volunteer group and professional contacts for community practitioners, we recruited nurses working in general practice, GPs, pharmacists, and care assistants working in general practice/community and older patients. Data were collected through an electronic survey distributed upon completion of the consent form. Participants were instructed to rate the ADRe Profile items on a 4-point ordinal scale [51], as follows:

1. Not relevant
2. Somewhat relevant
3. Quite relevant
4. Highly relevant

The 101 items on the ADRe Profile could score a maximum of 404 (if all items were rated as highly relevant) and a minimum score of 101 (if all items not relevant). Participants were also given an opportunity to comment on any items they deemed necessary for removal or addition, as well as to add any other comments in free-text format.

The CVI was calculated for each questionnaire item and represented the strength of agreement among participants regarding the relevance of the item (i.e., scoring 3 or 4 on the scale), as per Lynn [51]; Waltz and Bausell [52]; and Polit and Beck [53]. The multirater kappa statistic (ϰ) was calculated to determine the level of agreement beyond chance [54]. Kappa values were interpreted based on Landis and Koch's [55] criteria: > 0.80 indicating almost perfect agreement, 0.61–0.80 substantial agreement, 0.41–0.60 moderate agreement, 0.21–0.40 slight agreement and < 0 poor agreement, below the level of chance. The scale content validity ratio was calculated as a proportion of total items judged against content valid items and as the final average of individual CVI scores (as in [49, 56]). Free-text comments were analysed qualitatively.

### 2.6. Contrast Group Construct Validity

Construct validity was investigated through the comparison of contrast groups, with the expectation that their respective outcome measures would have different scores on a measurement scale [57, 58], taken here as the number of issues identified on the ADRe Profile. The contrast groups were constructed based on the quantity of prescribed medicines (no medicines vs. 5 or more medicines daily).

A total of 68 participants were recruited, 20 in the ‘no-medicines group' (65 years old or older, using no prescribed medicines (vitamins, nutritional supplements and moisturising skin preparations were not counted as medicines) and 48 in the ‘polypharmacy group' (65 years old or older, 5 or more long-term medicines prescribed by GPs). Information on the number of daily prescribed medicines for the ‘polypharmacy group' was extracted from the medical notes [35].

Patients were approached by clinicians and consented by researchers between May and December 2021.

All participants completed the ADRe Profile, either independently (via online platform or on paper) or with the researcher over the telephone. The sections on vital signs and medicines administration were omitted, leaving 57 items in the assessment. The Mann–Whitney statistic was used to test differences between the groups.

### 2.7. IRR

IRR was calculated based on self-administration followed by researcher-led administration of the ADRe Profile in person or via video link with 42 patients aged 65 or over prescribed at least 5 long-term medicines. Self-completion preceded researcher administration by 1–7 weeks.

The IRR of patient- versus researcher-completed ADRe Profiles was measured to ascertain the extent to which potential variability in records was attributable to random measurement errors [59, 60] and estimate the degree of agreement between patient and researcher evaluation of patients' symptoms. Additionally, the sensitivity and specificity of the ADRe Profile were calculated as (true positives)/(true positives + false negatives ) and (true negatives)/(true negatives + false positives ) [61, 62].

A minimum sample size of 50 participants was deemed sufficient for scenarios involving two raters and binary endpoints, to allow 2-sided testing against a null value of 0.00 for kappa with 80% power and alpha of 0.05 [63, 64].

For categorical ratings, the proportion of agreement between the two raters was calculated using the following equation: (number of agreements)/(number of agreements +  disagreements) [65]. Cohen's kappa (ϰ) was calculated as described in [66], using the equation below:

(1) $$\begin{array}{c} \varkappa =\frac{\text{proportion of agreement}-\text{probability of chance agreement}}{1-\text{probability of chance agreement}}. \end{array}$$

All participants received guidance on using the ADRe Profile, to improve rating consistency. To further minimise systematic error, brief written completion instructions and structured response formats with predefined answers were included.

## 3. Results

### 3.1. Cognitive Interviews

Analysis of the cognitive interview comments and suggestions led to 15 adjustments to the ADRe Profile. Eleven adjustments were made to the ‘Supporting information'; for example, ‘muscle cramps' [67] and ‘crossing and uncrossing legs when sitting' [68] were added to the list of manifestations of ‘Abnormal movements at rest'. Three adjustments were made in the ADRe Profile. For example, ‘More than 2 doses of prescribed medication missed over any period of 7 days in the last month' was replaced with ‘More than 2 doses of prescribed medication missed in the last 7 days', to increase clarity. One adjustment was made in the ‘How to' document. Detailed information regarding these adjustments is available in Supporting Information 1.

The cognitive interviews gave participants the opportunity to reflect on the ADRe Profile. All participants acknowledged the usefulness and need for such a tool. However, the healthcare professionals raised concerns regarding the time needed to complete the instrument and address any problems identified. In addition, the GP expressed apprehension regarding medicolegal vulnerability:

GP: ‘*But that also slightly fills me with a little bit of fear. If I end up with this questionnaire on my table that says the patient has got chest pain, shortness of breath, vertigo, skin problems, erectile problems, and mental health problems, and behavioural changes. I fear having all that information on the table, and now I am responsible for actioning this, all this information. You can't … un-know that information, you've got it there, documented in writing. You put it into the patient's notes, because we are told we have to record everything and suddenly it looks like we are ignoring* (smiles apologetically) *all these kinds of concerns that patients have got.'*

Other respondents were pleased to see potentially sensitive questions presented for discussion:

Pharmacist: *It is useful to include questions such as self-harm, physical aggression, violence, libido, and sex drive in the questionnaire, because such questions are rarely asked during medicine reviews. They are sensitive questions, which could be seen as judgemental if asked, but when included in a questionnaire, they can be seen as justified and it is easier to ask them. I don't normally ask about those things, and I bet you in your standard consultations no-one else would, so maybe there is actually an opportunity that someone would finally ask a question about that.*

The practicalities of introducing a holistic, comprehensive approach to monitoring polypharmacy were raised:

GP: *‘We certainly don't have an hour or half an hour or 20 minutes to do that. We should do, especially for our patients with polypharmacy and multiple, multiple problems but we just don't have that luxury, just because of the demand on our service, basically. So unfortunately, it should be a comprehensive chat about the medication, but it tends to be a quick ‘how are you doing on your medication, are you taking it? Yes/no. Any problems? No, OKay, well, you know, can you reduce your PPI* [proton pump inhibitor] *because it is causing you calcium absorption problems or whatever. OK, I'll try, thank you very much, bye bye.'*

Pharmacist: ‘*Specialist reviews (diabetic, asthma, blood thinning medicines) take place in the GP practice. Each of the reviews only deals with problems pertaining to a single speciality: if someone has,* for example*, a respiratory condition, diabetes and has 10 other tablets for different conditions, I don't touch the respiratory [medicines], I don't touch the diabetic* [medicines SIC], *because we have our respiratory nurse, our diabetic nurse, and it's about 20 minutes each to do those reviews properly'.*

This raises questions about the fragmentation of care and implies difficulties in finding the space/time for implementing the ADRe Profile.

A comprehensive overview of the cognitive interview feedback is included in Supporting Information 2.

### 3.2. CVI

Recruitment was by staff email to ∼150 academics in the Faculty of Medicine, Health and Life Science and other professional contacts. Fourteen participants were recruited to the CVI testing exercise: 5 GPs, 3 nurses, 2 pharmacists, 2 support workers and 2 patients. All participants completed the CVI survey online.

Among the 101 items in the ADRe Profile, two items (‘dizziness' and ‘falls') achieved unanimous agreement (14/14); 37 items met the generally accepted validity threshold, with a CVI of 0.78 or higher. The research team closely examined the 64 items with lower CVI values, leading to the removal of two items with CVI < 0.3 (‘girth measurement' and ‘are your drinks sugar-free?'). The calculated kappa values closely mirrored the CVI calculations, illustrating the diminishing effect of chance agreement with an increasing number of raters and the subsequent convergence of CVI and kappa values [69]. The scale-level index of the ADRe Profile was 0.67, with 37.4% of items demonstrating a significant level of agreement. Additionally, 85% of items had a CVI equal to or higher than 0.5.  Table 1 provides a summary of the results; full results can be found in Supporting Information 3.

The ‘*Medicines administration*' section of the ADRe Profile had the largest proportion of items with I-CVI > 0.78 (4/6), followed by the ‘*Vital signs*' section (8/18), ‘*Observations'* section (7/18), ‘*Reports/questions*' section (16/42), ‘*Summary*' (1/3) and ‘*Health promotion*' section (4/15). The calculated kappa values were very similar to the I-CVI calculations and demonstrated the diminishing effect of chance agreement with increasing number of raters and subsequent converging of I-CVI and kappa [69].

The traditional vital signs were rated highly, but, despite medicines causing weight changes and firm guidance in the British National Formulary [70], the item of ‘weight change' was not considered important. Only some posture and movement signs were rated highly, despite clinicians' reliance on pattern recognition. Pain, patients' views and health promotion were judged unimportant.

Participants' comments provided insight into their understanding of the ADRe Profile items. The mean time spent completing the CVI survey was 12 min (SD = 11.73). Given the instrument's complexity, some items may not have been fully understood. For example, CVI participant 2523 (a health care support worker) stated:

*‘The section on medicines management, don't [sic] make much sense to me. Looking at the first question ‘(are) any tablets crushed or broken?' What exactly are you asking here?'*.

This has likely influenced the CVI results. Another respondent's comment confirmed the need for the ADRe Profile and patient safety training:

‘*we normally do not do such a structured and detailed review I would say, so that means that people haven't been previously trained on the ADRe Profile documentation*' [a pharmacist].

### 3.3. Contrast Group Construct Validity

Sixty eight participants contributed data. The ‘–no-medicines group' and the ‘polypharmacy group' participants had comparable demographic characteristics: Median (IQR) ages were 68.50 (65.25–74.00) and 73.50 (69.00–78.00); 45% and 44% were female. Group differences in the numbers of problems identified on the ADRe Profile are summarised in Supporting Information 4. There was a significant difference in the median number of problems stated (*U* = 870.50, *p* < 0.001).

Certain ADRe Profile items, such as ‘tongue tremor' and ‘self-harm', were not reported by any participants in either group. Most items (*n* = 27) were not reported by participants in the ‘no-medicines group' but were present in the ‘polypharmacy group'. Examples include *‘feet shuffling'* (*n* = 11 [23%]) and *‘problems with balance'* (*n* = 18 [38%]). Several issues were encountered by participants in both groups, but the proportion of participants experiencing these issues in the ‘no-medicines group' (*n*_1_) was lower than in the ‘polypharmacy group' (*n*_2_). Examples include *‘pain'* (*n*_1_ = 7 [35%] vs. *n*_2_ = 37 [77%]), indigestion (*n*_1_ = 2 [10%] vs. *n*_2_ = 20 [42%]) and *‘sleep problems'* (*n*_1_ = 5 [25%] vs. *n*_2_ = 15 [31%]).

### 3.4. IRR

Most (42 of 48) eligible participants (from 5 GP practices) returned their self-completed ADRe Profiles. One of the six noncompleters struggled to read even large print due to advanced cataracts, two disliked completing paper forms, and three participants postponed/neglected form completion. IRR was calculated for 78 questions presented in both the self-administered and researcher-administered ADRe Profile. Vital signs and the patient summary were not included in the IRR testing. Agreement was complete for factual statements, for example visits to dentists, and lower for less precisely defined questions, for example skin or respiratory problems. And, 10 of 78 items had complete agreement. The findings are summarised in Table 2, and kappa results per category can be found in Supporting Information 5.

The simple percentage agreement ranged from 69% to 100%, with a mean of 91.45% (SD = 6.92) and a median of 93 (25th percentile: 86%, 75th percentile: 97%). The aggregate kappa values indicated substantial agreement beyond chance. The full IRR report by item may be found in the Supporting Information 6. Given the recognised effects of prevalence bias and natural fluctuations of signs and symptoms over 1–7 weeks [71], these values were considered satisfactory and denote significant concordance in the service user and researcher ratings of items in the ADRe Profile. Sensitivity and specificity, unaffected by problem prevalence, were notably high (with mean sensitivity and specificity for the researcher rating at 85% and 90%, respectively).

## 4. Discussion

Evaluation of instrument validity and reliability contributed to development of the ADRe Profile for primary care use (see also: [72]). Validity and reliability were supported through complementary mixed measures: Construct validity was indicated by the CVI of 0.67 and cognitive interviews; discriminant or contrast validity indicated a difference in reports between service users not prescribed regular medicines and those prescribed five or more (*U* = 870.50, *p* < 0.001); IRR was demonstrated for all items (mean kappa 0,71, range [0.31–1.00]).

### 4.1. ADRe Profile Validity and Reliability

Content validity was assessed by confirming that the ADRe Profile items reflected the notion of ADRs and suboptimal medicines management, using expert ratings (CVIs) and cognitive interviews to establish the CVI [51, 69]. Construct validity was evaluated by implementing the contrast group approach, comparing individuals taking no medicines versus those taking five or more, to assess whether the ADRe Profile could effectively distinguish between groups with expected differences in ADR-related health issues [57]. This use of ‘known groups', where only one group could possibly have ADRs, offers an external measure of discriminant or criterion validity [73].

The assessment of validity and reliability properties was approached within a continuous framework [59] rather than as dichotomous outcomes. This approach implies that the accumulation of evidence increases confidence in the instrument's quality and the value of the data it generates.

The cognitive interview testing confirmed that most of the ADRe Profile items were clearly understood by the target population and appropriate for their situation. Testing of previous versions of the Profile [32] resulted in gradually diminishing numbers of implementable suggestions, indicative of progressive refinement of the instrument.

Favourable evaluations with cognitive interviews were corroborated by findings from the construct validity evaluation. Consistent with existing literature [57, 58], administering the instrument to contrast groups elicited divergent scores. The ADRe Profile identified significantly more problems among people using 5 or more medicines compared with those who had no regularly prescribed medicines.

Regarding content validity, just over a third of the questionnaire items met the generally accepted validity threshold (CVI of 0.78 or above), contrasting with previous findings of high scale CVI of 0.99 [32], or 93 out of 102 items rated as of the highest importance to delivery or outcomes of care [31]. It is unlikely that small differences in ADRe Profile versions account for this discrepancy, suggesting potential variations in raters' perspectives. While explanation for all items is included in the Supporting information part of the ADRe Profile, busy raters may not have time to read this resource sent to them in the ∼12 min they allotted to completing the CVI; therefore, ratings may not have been fully informed.

Previous ADRe Profile effectiveness studies, which consistently demonstrated clinical benefit [26, 29, 31, 33, 34], and literature and clinical guidelines support the inclusion of all ADRe Profile items. User judgement of content validity may depend on knowledge and experience. Knowledge of pharmacology and, possibly, ADRe Profile training, may be crucial in future use.

The ADRe Profile demonstrated consistency across patient self-report and nurse researcher completion, as evidenced by high agreement and substantial aggregate kappa values during IRR testing, and high sensitivity and specificity results, comparable to previous testing findings [27, 32].

Although some ADRe Profile items are measurable (e.g., blood pressure or weight) or observable (e.g., gait or swelling), others rely on service users' reports of symptoms (e.g., pain, tiredness or forgetfulness). This poses challenges associated with the reliability and stability of self-reported data [74–77]. While literature specifically examining self-reporting health issues yields variable results [78–80], the IRR determined that participant ADRe Profile responses are sufficiently stable for meaningful use in clinical practice. Reliability testing empowered patients to recognise some clinical problems that had developed insidiously over some years and empowered them to seek help. For example, patients were helped by recognition that tremors plus abnormal movements may be pathognomonic of drug-induced Parkinsonism in patients using gabapentinoids, or that bleeding may indicate a need to reduce doses of anticoagulants.

### 4.2. The Need for H–gh-quality Instruments Identifying ADRs

Questionnaires designed to identify ADRs should include a combination of closed and open questions, providing opportunities for service users to add comments and requests. Relying solely on open-ended questions such as: ‘Do you experience any side effects?' assumes that patients have a comprehensive understanding of ADRs' symptoms. Therefore, questionnaires must be comprehensive enough to detect all or most ADR signs and symptoms. Since many commonly prescribed medicines each have many potential ADRs, for example there are ∼100 ‘undesirable effects' for risperidone [81] and ∼80 for gabapentin [82], it is difficult to reduce the list of potential problems whilst retaining a focus on safety. Healthcare professional respondents indicated that while the ADRe Profile is comprehensive, it is also perceived as too long and identifies clinical problems that professionals need to find time to manage. This may pose challenges for completion within the constraints of U.K. NHS practice. However, this downside is likely to be balanced by the clinical gains stemming from thorough ADR identification and the early resolution of issues [27, 83].

The WHO ([84], p. 8) notes that current guidelines focus on single diseases, reducing their effectiveness. As a pharmacist respondent (P1) noted, the ADRe Profile covers a wider range of conditions, and must consequently take the form of a longer instrument requiring significant completion time. The ADRe Profile includes problems potentially related to a range of medicines and offers decision support relating to commonly prescribed medicines. It meets the WHO criteria for management of polypharmacy in that it implements medication reviews in collaboration with the patient ([85], p. 10). However, as our interviewees noted, close engagement with patients poses considerable difficulties given the current demand and resource pressures affecting U.K. primary care, and these will be difficult to overcome in the short term, without managerial commitment to reduce the number of unplanned hospital admissions due to ADRs.

### 4.3. Costs

Decision aids, such as the ADRe Profile, enhance professionals' perceptions of knowledge and judgement, but extend consultation time, particularly on first use [86, 87]. Costs of this extended patient contact are estimated as £14 for 15 min of a pharmacist's time, plus £4 for 10 min for a support worker to record vital signs; where an additional, unscheduled GP review is needed, this costs £32 [88]. Many of the problems addressed by ADRe Profile administration (e.g., relief of pain, dyspnoea or sedation) benefit patients but are difficult to cost. Many patients had several problems that could lead to hospitalisation. The quantifiable economic benefits of the ADRe Profile may be related to identification of these problems and prevention of admissions [29]. For example, each admission due to a fall, without a prolonged stay, costs ∼£18,000 [89]. Therefore, the costs of the ADRe Profile would be recouped were it to prevent 1 falls' admission for each 250–730 administrations. Simultaneously, if pre-emptive action prevents bleeding escalating to an upper gastrointestinal haemorrhage, costing ∼£3000 [90], the costs of 40–120 ADRe Profile administrations would be recouped.

### 4.4. The ‘ADR Problem'

The ‘ADR problem' continues to cost lives, cause preventable suffering and strain health services' resources [84] but has not been solved by existing strategies, such as medication reviews [10, 11, 14–16, 31]. Reactive, post hoc, voluntary reporting of serious ADRs (such as the U.K. Yellow Card scheme), whilst essential for postmarketing pharmacovigilance, does not benefit affected patients. Reviewers estimate voluntary reporting overlooks 94% of events [91], whilst is practice, < 1% of drug-induced GI haemorrhages are reported in the United Kingdom [92]. The role of pharmaceutical manufacturers in analysing [93] and disseminating ADR information may be suboptimal [94]. Accordingly, comprehensive instruments need to be incorporated into routine practice. Patient-reported outcome questionnaires, with decision support, are a useful way of gathering comprehensive ADR data to complement medicine reviews.

Currently, most patient-reported questionnaires focus on specific medication classes, mostly mental health medicines, and lack decision support, for example the Glasgow Antipsychotic Side-Effect Scale [95], or the Maudsley Side-Effects Scale [96]. Few instruments target commonly prescribed medicines and, where they do, they may not be comprehensive [97]. Moreover, many patient-reported questionnaires lack critical quality indicators: A systematic review identified 19 patient-reported outcome instruments, but only half had been assessed for validity (50%) and even fewer for reliability (< 50%), with very few tested in trials [98]. Before clinical implementation, instruments must be supported by publicly funded evidence of reliability, validity, feasibility, and clinical effectiveness: A template is offered in this paper.

## 5. Strengths and Limitations of This Study

This work builds on earlier reports of reliability and validity of the ADRe Profile [27–33]. Combining the methods of testing offers an overview of instrument testing and leaves an overall impression as to the value of the ADRe Profile. The feasibility of administering the ADRe Profile is reported in tandem with this paper.

The ADRe Profile does not generate a scale or a score; rather, it lists individual problems. To our knowledge, there are no ‘gold standard' comprehensive ADR scales for polypharmacy to which the ADRe Profile might be compared to test criterion validity [73]. It was not possible to verify all participants' reports of symptoms to external testing. Traditionally, clinicians rely on patient self-report of symptoms [99]. Earlier ADRe studies have shown how acting on these self-reports reduced symptoms, such as pain or aggression. Reports of vision difficulties were externally validated when opticians prescribed spectacles; similarly, pain symptoms were externally validated when dentists extracted or filled teeth [28, 29].

The relatively low numbers of participants for some tests reflects the iterative nature of ADRe Profile refinement and available resources. However, similar findings to previous work indicate the practical adequacy of the ADRe Profile [100].

The validity and reliability testing had both strengths and limitations. The cognitive interviews adhered to recognised frameworks in planning, conducting, analysing and reporting [41, 47, 101]. However, the sample size was small and the conventional qualitative indicator for data collection cessation—data saturation—was not pursued. It is, therefore, possible that further interviews would collect data not captured in this study.

The construct validity testing in this study is specified relative to the construct that people who take 5 or more regular medicines exhibit more ADRs than people who do not. However, the aetiology of health and wellbeing problems is often multifactorial, making it challenging to distinguish between issues stemming from ADRs, undertreated health conditions, or symptoms of physiological ageing.

The limitations of IRR testing were mostly attributable to imperfect adherence to some of the scenario assumptions in Cohen's kappa determination, and conflation with test/retest calculations. Firstly, the time lapse between patient and researcher completion of the ADRe Profile may have triggered some of the issues typically affecting test–retest reliability assessment, such as the carryover effect of the second response being affected by the person's initial response, or real change in the observed problems over time [102]. This natural fluctuation in clinical signs and symptoms precluded formal (and complex) statistical assessment of test/retest reliability, Secondly, while the raters are assumed to operate independently [66], the second rating in this study occurred in a conversation between the nurse and the patient (Rater 1 and Rater 2).

## 6. Conclusion

Addressing the ADR problem requires multifaceted approaches, including the development and validation of comprehensive patient-reported outcome decision support questionnaires, alongside the integration of WHO-endorsed practice for managing polypharmacy. By addressing these challenges, healthcare systems can enhance patient safety and optimise the allocation of resources, ultimately mitigating the adverse impacts of ADRs on public health.

The ADRe Profile is suitable for use with older patients in primary care who live at home. Intended users of the instrument had a good understanding of the meaning of ADRe Profile questions and were able to process and record meaningful answers to questions. ADRe Profile responses were sufficiently reliable to be used as a basis for further investigations, prescriber referral and clinical actions.

This paper has described how the validity and reliability of the ADRe Profile were assessed. As part of a wider study, it laid the groundwork for subsequent feasibility testing and a RCT. This approach may serve as a methodological precedent or template for future studies seeking to evaluate the efficacy of novel instruments in healthcare settings.

## 7. Implications for Nursing Management

To ensure that avoidable problems related to prescribed medicines are not overlooked, managers should facilitate introduction of patient-centred symptom monitoring, with decision support. The safety of older patients prescribed multiple medicines necessitates using a comprehensive instrument, such as the ADRe Profile. Managers should be confident that reliability and validity data are available for instruments adopted.

## Figures and Tables

**Table 1:** Overview of the ADRe Profile content validity index results.

ADRe section	Section S-CVI	Modified kappa—section mean
Vital signs	0.62	0.62
Observations	0.69	0.69
Reports, questions	0.72	0.72
Prevention and health promotion	0.50	0.50
Medicines administration	0.83	0.83

**Table 2:** Kappa frequency statistics, overall and per section.

ADRe profile items (*n* = 78)	Valid	Missing	Mean kappa value	Std. deviation	Range (min–max)
Overall	70	8	0.71	0.17	0.69 (0.31–1.00)
Observation section	17	1	0.72	0.16	0.62 (0.31–0.93)
Reports section	38	3	0.73	0.16	0.62 (0.38–1.00)
Health promotion section	12	1	0.69	0.22	0.68 (0.32–1.00)
Medicines section	3	3	0.60	0.05	0.10 (0.55–0.66)

## Data Availability

All relevant data are included in this manuscript or the Supporting Information. Further details are available from the author on request.

## Supporting Information

Supporting Information 1 Supporting Information 1: Cognitive interviews—suggested changes to the ADRe Profile and Supporting information.

Supporting Information 2 Supporting Information 2: Cognitive interview participant comments.

Supporting Information 3 Supporting Information 3: Content validity index by ADRe Profile sections.

Supporting Information 4 Supporting Information 4: Between-group differences in problems experienced.

Supporting Information 5 Supporting Information 5: Inter-rater reliability—kappa results per category.

Supporting Information 6 Supporting Information 6: Full results for inter-rater reliability.
