# Supplementary material for: Reducing Adverse Drug Reactions for Older People in the Community: Evaluating the Validity and Reliability of the ADRe Profile
Source: J Nurs Manag. 2025 May 14;2025:9921349. doi: 10.1155/jonm/9921349 (PMC12094870; doi:10.1155/jonm/9921349)
Supplement: Supporting Information 1 — Cognitive interviews—suggested changes to the ADRe Profile and Supporting information. [file 9921349.f1.docx]

Supplementary material 1: Cognitive interviews - suggested changes to the ADRe Profile and Supplementary information

Abbreviations: Ph – pharmacist, GP – general practitioner, N – nurse, SU – service user (patient)

The changes highlighted in yellow affect the ADRe Profile items, changes highlighted in green affect the supporting information

Problems identified by more than one participant are underlined.

| Item | Suggestions | Who/ Date | Initial response | Final response |
| --- | --- | --- | --- | --- |
| Hand tremor affecting drinking OR eating OR fastening buttons/zips  Answer categories: No, Yes, Worse | Clarity: It is not clear whether ‘worse’ refers to today, this week or this month. | Ph  23/04/21 | Include the time frame for ‘worse’ option as ‘since last review’ in the How to document. | Depends on the context. Text added to ‘How to’ document:  ‘Worse’ is usually taken as the patient’s perception of an overall trend. |
| Restlessness or pacing | Repetition: This item is very similar to ‘Abnormal movements at rest’, explored earlier. | Ph  23/04/21 | No action in the ADRe Profile, both items are relevant as on the first occasion symptoms are observed, and on the second occasion the service user is asked about their feelings.  Changes in the supporting information: ‘Does the service user feel restless or unable to relax?’ ‘Are they aware of any abnormal movements in their face or extremities?’ | Text copied into the supporting information. |
| More than two doses of prescribed medication missed over any period of seven days in the last month. (including refusal)? | Clarity: Confusing sentence, needed to read several times to understand. | Ph  23/04/21  SU  15/05/21 | Substitute with: ‘More than 2 doses of prescribed medication missed in the last 7 days.’ | Text in the ADRe Profile changed to:  ‘More than 2 doses of prescribed medication missed in the last seven days.’ |
| Abnormal movements at rest: Any involuntary movements as if chewing or sucking? Wringing movements of fingers or feet? Sudden jerking movements? Does service user feel restless? Is s/he pacing? | Instructions: People who are not sure about what abnormal movements at rest to observe for and rely on the supporting information will tend to use the list as exhaustive, ie only observe for listed symptoms. | Ph  23/04/21 | Add muscle cramps (Datapharm, n.d.), crossing and uncrossing legs when sitting (Pringsheim et al., 2018).  Substitute ‘Does service user feel restless?’ as this is not observable, with ‘Does service user appear restless?’ |  |
| Hair loss: If possible, discuss with same gender nurse | Assumptions:  It is not clear why this should be discussed with the same gender nurse. | Ph  23/04/21 | This is subject to opinions. | No change in the ADRe Profile – there may be generational attitudes to hormonal balance. |
| Confusion: check reports of ‘feeling like a zombie’. | Assumptions:  Very old service users may not be familiar with the word ‘zombie’. | Ph  23/04/21 | No change. Victor Halperin’s White Zombie film first appeared in 1932 (Luckhurst, 2015). | No change in the ADRe Profile. |
| Non-verbal pain indicators present? – lack of supporting information | Instructions:  Supporting information listing examples of the non-verbal signs of pain would add clarity. | Ph  23/04/21  GP  29/04/21 | Add the following guidance:   \| Facial expressions \| Grimacing, clenched jaw, wrinkled forehead \| \| --- \| --- \| \| Body movements \| Restless or rigid posture \| \| Verbal response \| Moaning, groaning, crying \| \| Physical signs \| Pallor, altered breathing, sweating, change in vital signs \|   (based on Varndell et al., 2017)  Consider assessment with PAINAD tool (<http://dementiapathways.ie/_filecache/04a/ddd/98-painad.pdf>) or Doloplus-2 (<https://prc.coh.org/PainNOA/Doloplus%202_Tool.pdf>), as recommended by the UK national guidelines (Schofield, 2018). | To include a comprehensive list would take a lot of space, so links to pain assessment tools will be included instead:  Added  ***‘If a problem or concern is detected:***  Consider assessment with PAINAD tool (<http://dementiapathways.ie/_filecache/04a/ddd/98-painad.pdf>) or Doloplus-2 (<https://prc.coh.org/PainNOA/Doloplus%202_Tool.pdf>), as recommended by the UK national guidelines (Schofield, 2018).’ |
| High salt intake? | Instructions:  Question guidance would be more comprehensive if it also included information on low sodium high potassium salts. | Ph  23/04/21 | Add the following to the High salt intake supplemental information: Caution: low-sodium salt may be unsuitable for patients with hyperkalaemia.  Add the following to the ECG supplemental information: ‘Potassium concentrations may be raised by dietary supplements, including low-sodium salts, ACE inhibitors, NSAIDs, beta blockers or dehydration, kidney disease, severe illness or, occasionally, unusual diets.’  Add the following (in red) to Swallowing difficulties supplemental information: ‘Sudden onset of dysphagia may be related to oesophageal injury and should be reported; risk factors include: NSAIDs, bisphosphonates, iron preparations, potassium salts (included in low-sodium salts), some antibiotics.’ | Added |
| Sun exposure Is sunscreen of 4 stars and high factor available? | Instructions: Participant did not understand the meaning of four stars | Ph  23/04/21 | Add the following to the supplemental information: ‘Sunscreen should be high factor (15+) and high stars (at least 4) to ensure protection from both UVB and UVA light.’ (British Association of Dermatologists, n.d.) | added |
| Medicines bought without a prescription, include herbal preparations? | Professional experience shows additional probing needed for the patient to answer this question. | Ph  23/04/21 | Include: ‘Tip – ask about any preparations that were bought in the supermarket, pharmacy or the health shop.’ | Added  ‘Consider checking: any medicines bought at a chemist, supermarket or other shops?’ |
| Alcohol over-use (>2 drinks on 1 occasion) [optional question]? | This should not be an optional question. | Ph  23/04/21 | No change. It is on the health professional’s judgment whether they will ask this question. | No change.  There is a risk of causing offence e.g.  some faith groups. |
| Various locations | Out-of-context symbols appearing in the text in several locations. | Ph  23/04/21 | Correct the electronic version to ensure smooth flow of text. | Corrected. |
| Tongue abnormal movements or tremor | Relevance: Participant unfamiliar with this symptom | GP  29/04/21 | No action in the ADRe Profile, drug-induced tardive dyskinesia commonly presents with abnormal facial and tongue movements or tremor (Frei et al., 2018; Ward & Citrome, 2018) | No change |
| ANY bleeding/bruising/nosebleeds | Instructions: The timeframe for reporting the problem is not specified, the participant would assume the timeframe to be since starting on medications. | GP  29/04/21 | No action in the ADRe Profile, the timeframe would depend on the context and professional judgment of the health professional, it may be from the start of new medications, since the last review, or within the last month. | Agree |
| Acne/hirsutism/*herpes simplex* (cold sores) | Jargon: Some health professionals may not be familiar with the term ‘hirsutism’ | GP  29/04/21  N  05/05/21  SU  15/05/21 | Add the following explanation in the supporting information: ‘Look for acne, excessive growth of dark hair on face, neck, chest or thighs in females and cold sores.’  It was noticed during the search that hirsutism is incorrectly spelled as hirsuitism in the electronic version of the ADRe Profile. Suggested action is to correct the misspelling.  In the SERVICE USER VERSION of the ADRe Profile, the item will be substituted with Acne/excessive hair growth/cold sores. No change in the version intended to be used by healthcare professionals. | Added:  ‘Look for acne (spots), excessive growth of dark hair on face, neck, chest or thighs in females and cold sores around the mouth.’  Spelling corrected in electronic version  Following discussion with the supervisory team, it was decided that only a single version of the ADRe Profile would be made. The language was adjusted to avoid or explain jargon and the person was kept neutral or second person. |
| Physical violence to people or objects | Forward question, the service user may not feel comfortable with answering this question truthfully. Potential social desirability bias. | GP  29/04/21 | No change. While the comments are valid, no suitable alternative has been found. | No change. |
| Low energy, weakness, fatigue, apathy | Jargon: Some health professionals may not be familiar with the meaning of ‘apathy’ | GP  23/04/21 | No change or add the following to the supporting information: ‘Reduction in self-initiated/goal-directed activity and motivation’ (Le Heron et al., 2019; Chong, 2020). | No change. The new phrases are more complex. |
| Sedation/excessive sleep (>2 hours over normal) | Instructions: Unclear period over which to assess, the participant would have understood over 24 hours. | GP  29/04/21 | No change. The supporting information already states ‘sleeping 2+ hours in the daytime’ | No change. |
| Discretionary question: Reproductive system  e.g. breast discomfort, erectile disfunction, change in libido (sex drive) | It is not clear why this question is labelled as discretionary, while other questions (eg. self-harm) are not. Also: it is not specified what clinical situations should trigger the question, if discretionary. Time-pressed health professionals may just skip all questions that are not compulsory. | GP  29/04/21 | Valid comments.  Should self-harm be discretionary? | No change.  This question must be discretionary in research. It may be discussed later whether it should be discretionary in practice. |
| **Heart** Chest pain | What are the medical/legal expectations of the healthcare professional who identifies chest pain? Motivation to ‘cover one’s back’ may lead to increased referrals to GPs. | 29/04/21 | The supporting information already specifies further actions. This comment illustrates the need to cover emergency and urgent situations in the teaching package for users of the ADRe Profile. | No change to the ADRe Profile or supporting documentation. Teaching package will include urgent and emergency situations. |
| High salt intake? | Instructions: The quantification of normal salt intake as 4.2-6.0g/day may be difficult to visualise for some service users. | GP  29/04/21  SU  15/05/21 | Add the following in red to the information sheet:  Is salt intake excessive? Daily recommendation is 4.2-6.0 g/ day salt (NICE 2019a) – this is about a teaspoon of salt. This includes medicines, salt added during cooking or once the meal is prepared, and packaged food e.g. crisps, ready meals. Check sodium content of medicines and product labels of packaged food. Particularly important for patients prescribed NSAIDs or corticosteroids or using antacids or effervescent preparations. If lithium is prescribed, ensure salt intake does not fluctuate (Taylor et al 2018).  In the SERVICE USER VERSION of the ADRe Profile, Supporting information will be substituted by: ‘Do you regularly add salt to your food? Do you eat a lot of crisps and ready meals? | Actioned. |
| **Sun exposure** Is **sunscreen** of 4 stars and high factor available? | Instructions: Availability does not imply that it is used. | GP  23/04/21 | No change, the intention is clear. | No change. |
| N/A | Palpitations should be added as a potential symptom of ADRs. | N  05/05/21 | Palpitations could be included temporarily while it is not possible to measure the service users’ vital signs. | Palpitations appears with chest pain, irregular rhythm etc.  The terms are poorly understood and could cause alarm.  It was removed for these reasons. |
| N/A | Service user’s allergies should be added to the ADRe Profile. | N  05/05/21 | No action: ADRe Profile is designed to be used in conjunction with the MAR chart, allergies are included in the MAR chart, and there is no need for duplicating the information. | This is included in the summary for pharmacists.  No need to appear twice. |
| CNS - Any convulsions (even if epileptic), | Jargon: Service users may not respond to the jargon; simpler language would be preferable. | N  05/05/21 | Participant suggested the following explanation to the service users: Have you had any fits, faints, falls or funny turns? | Convulsions / seizures appears as item.  Falls are separate. ‘Funny turn’ is too vague. Fits is not PC, and faints are likely cvs.  No change has been implemented. |
| Falls | Further probing questions needed to understand the nature of falls. | N  05/05/21 | Possible addition: If the problem detected, ask how many falls the service user had, was it a fall or a stumble? were they witnessed, how did they happen – tripped over something? Result of dizziness? Light-headedness? | No change, this information is already covered under dizziness:  ***Please review information on assessing:***   - Do you ever feel light-headed, particularly on standing for a long time or suddenly? - Did you feel dizzy while standing? Getting up from lying down? Getting up from a chair? - How many times have you felt unsteady while undertaking normal activities (such as walking) in the last month?   Any falls or stumbles? **Recheck BP lying and standing (vital signs, above).** |
| Headache/migraine | Suggested further probing to find out where and how they are affecting the service user. | N  05/05/21 | Possible addition: If the problem is detected, enquire about the nature of the headache/migraine | No change, we already have: Explore onset of headache in relation to prescription regimen. |
| Appetite/taste changes | Further probing suggested to understand the reasons for appetite/taste changes. | N  05/05/21 | Possible addition: It the problem is detected, enquire about the nature of the changes. | No change, hcps will know to do this. |
| Hand tremor affecting drinking OR eating OR fastening buttons/zips | Participant not sure how to indicate if he only had one of the symptoms | SU  15/05/21 | No action in the ADRe Profile, new version already has a tick next to each item (circle on paper copy).  Any one of these gets a ‘yes’. | No change. |
| Posture abnormal | Some people may not be aware of their abnormal posture, so may answer incorrectly | SU  15/05/21 | No action in the ADRe Profile, this item should be observed when possible. | No change. |
| Feeling the cold / cold to touch | Participant not sure which of the questions to answer | SU  15/05/21 | No action in the ADRe Profile, new version already has a tick next to each item (circle on the paper). | No change. |
| Rash (± itching) | Instructions: Time frame is not specified | SU  15/05/21 | No action in the ADRe Profile, recent or persistent rash should be reported. | No change. |
| Sweating | Sensitivity: Participant felt that some people might feel offended by the word and suggested ‘perspiration’ instead | SU  15/05/21 | No action, this was an isolated comment and was not supported by any other users of the Profile. | No change. |
| Other skin abnormalities (e.g. change in skin colour) | Time frame not included. | SU  15/05/21 | No action, recent or persistent abnormalities should be reported. | No action. ADRe used at different times. |
| **CNS** Any convulsions (even if epileptic) | Acronym/jargon used. | SU  15/05/21 | The following changes were made in the ADRe Profile: CNS will be substituted with ‘central nervous system’ or omitted (preferably). No change in the version intended to be used by healthcare professionals.  Participant thought the term ‘convulsion’ was clear and did not need substituting with ‘seizure’. | CNS was followed by central nervous system. |
| Sleep problem/insomnia | Participant appeared to misunderstand the term. | SU  15/05/21 | The following changes will be made in the ADRe Profile, insomnia will be followed by (inability to sleep). | Actioned. |
| **GU tract** Urination problems / incontinence / burning / discomfort on urination / UTI | Acronyms/jargon used. | SU  15/05/21 | The following changes will be made in the ADRe Profile, omit ‘GU tract’ and substitute ‘UTI’ will full version. | Actioned. |
| Meals missed or left unfinished more than once a day?  Two or more meals (1 cooked) eaten daily on 6 of last 7 days? | Participant thought the questions were very similar. | SU  15/05/21 | No change, the first question asks about inability to finish the meal, and the second about availability of the meals. | No change. |
| Behaviour problems | People may not be able to distinguish between normal emotions of anger and problematic behaviour | SU  15/05/21 | Possible change: In the SERVICE USER VERSION of the ADRe Profile, use the following explanation in the Supporting information: Do you or other people feel that your behaviour is not socially acceptable? Does your behaviour negatively affect your relationship with other people? Are you sometimes worried about how you behave?  No change in the version intended to be used by healthcare professionals. | No change. As indicated, no definitions of behaviour problems in adults are available.  Single version of the ADRe Profile will be kept. |
| All items | Clarity: Phrasing the items as complete questions would be clearer for the service users. | SU  15/05/21 | Possible change: In the SERVICE USER VERSION of the ADRe Profile, clear heading of: ‘Do you experience any of the following symptoms’ will precede the list of items.  In the SERVICE USER VERSION, the Supporting information will be re-phrased to address the service user and provide additional information about the item. Exclamation mark information will be removed as this is intended for the healthcare professional.  No change in the version intended to be used by healthcare professionals. | A decision was made to keep a single version of the ADRe Profile. The language was made neutral or in the second person, to enable independent service user use of the Profile.  Addition on the ADRe Profile: ‘Click on the relevant space to indicate presence or absence of problems. Provide extra information in the free comments box if problem detected or worsening.’ |
