# Supplementary material for: Reducing Adverse Drug Reactions for Older People in the Community: Evaluating the Validity and Reliability of the ADRe Profile
Source: J Nurs Manag. 2025 May 14;2025:9921349. doi: 10.1155/jonm/9921349 (PMC12094870; doi:10.1155/jonm/9921349)
Supplement: Supporting Information 2 — Cognitive interview participant comments. [file 9921349.f2.docx]

Supplementary material 2: Cognitive interview participant comments

Cognitive interview results

**Cognitive interview 1**

The first cognitive interview was performed on the 23^rd of^ April 2021 with a pharmacist (participant code = CIP). CIP works as a GP cluster pharmacist and is also a pharmacist prescriber. The research supervisor (SJ) was also present during the first interview as an observer, as part of standard cognitive interview training. The interview took 67 minutes.

Findings

The cognitive interview with CIP illuminated several potential issues in the ADRe Profile items, a number of improvements to the supporting information section, as well as inaccuracies in the “How to” document. The findings are summarised in table 00, other comments and thoughts pertaining to evaluation of the ADRe Profile, and its implementation are summarised below.

Other comments

The participant highlighted the need for training to use the ADRe Profile, as the instrument was unfamiliar to her.

The respondent commented on the advantages of having the ADRe Profile online, including time savings and potential integration with other electronic patient data. Despite this the respondent’s personal preference was to complete the questionnaire on paper, due to perceived loss of rapport with the service user when using an electronic device.

It is useful to include questions such as self-harm, physical aggression, violence, libido, and sex drive in the questionnaire. According to the respondent’s experience, such questions are rarely asked during medicine reviews. They are sensitive questions, which could be seen as judgemental if asked, but when included in a questionnaire, they can be seen as justified and it is easier to ask them.

CIP also shared an insight that in her experience, patients correct response is sometimes triggered by specific wording, for example, asking whether the patient buys over-the-counter medicines results in a negative response, but asking the patient whether they buy any medicines or supplements from a health store or Boots, the patient’s memory is triggered, and they list the over-the-counter medicines they use.

The length of the questionnaire was to be an often-repeated comment. While acknowledging the comprehensiveness of the instrument, CIP thought that the practicalities of constant time pressure prohibited its use. The respondent described the specialist reviews (diabetic, asthma, blood thinning medicines) that take place in their GP practice. Each of the reviews only deals with problems pertaining to a single speciality. This practice raises questions about the fragmentation of care and implies difficulties in finding the space/time for implementing the ADRe Profile.

**Cognitive interview 2**

The second cognitive interview was performed on the 29^th of^ April 2021 with a GP (participant code = CIG). CIG works as a GP in a practice without a cluster pharmacist and is also responsible for yearly medicine reviews. The interview took 33 minutes. “How to” document was not discussed due to time pressures.

Findings

CIG interview provided insight from a different professional viewpoint. It also illuminated several potential issues in the ADRe Profile items and the supporting information section. The findings are outlined in table 00, other comments and thoughts pertaining to evaluation of the ADRe Profile, and its implementation are summarised below.

Other comments

In the GP participant’s opinion, the ADRe Profile would be a useful instrument to implement, especially for older service users with polypharmacy. The participant acknowledged polypharmacy as a persistent problem in his place of work. Current practice in the participant’s workplace was for GPs to review medicines at least once yearly. A monitoring computer system did not allow the receptionist to re-issue repeat prescriptions without the yearly review. At the same time, GPs had control over the time period of the review, so the review may be set for 3 months or one week – which was the participant’s practice when first starting certain medicines, for example, SSRIs.

Continuing with the time pressures issue, the participant noted that GPs would not have the ‘luxury’ of doing a comprehensive medicine review and went on to describe the typical review procedure: *‘We certainly don’t have an hour or half an hour or 20 minutes to do that. We should do, especially on our patients with polypharmacy and multiple, multiple problems but we just don’t have that luxury just because of the demand on our service, basically. (interviewer: yeah). So unfortunately, it should be a comprehensive chat about the medication, but it tends to be a quick sort of how are you doing on your medication, are you taking it, yes, no, any problems, no, OK, well, you know, can you reduce your PPI because it is causing you calcium absorption problems or whatever, OK, I’ll try, thank you very much, bye bye.’* An added common problem appears to be that patients often try to utilise the little time they have with the GP to discuss other health issues which are not related to the medicines review.

Considering the ADRe process where other health professional gathers the data, the pharmacist reviews the information and the GP is then sent recommendations, CIG thought this would make it easier for GPs from the time/resources point of view, yet voiced another concern regarding accountability: *‘But that also slightly fills me with a little bit of fear if I end up with this questionnaire on my table that says the patient has got chest pain, they’ve got shortness of breath, they got vertigo, they got skin problems, they ‘ve got, you know they’ve got erectile problems, and mental health problems, and behavioural changes, you know, I fear having all that information on the table, and now I am responsible for actioning this, all this information, OK. Because a lot of it may be to do with polypharmacy but a lot of it may be just due to them being old, but it’s… you can’t … once you … you can’t … un-know that information, you’ve got it there, documented in writing, you put it into the patient’s notes, because we are told we have to record everything and suddenly it looks like, you know, we are ignoring, we are ignoring* (smiles apologetically) *all these kind of concerns that patients have got.’*

Another point that was evident from the GP participant’s comments was that in his opinion, a lot of older service users would answer many ADRe Profile items with ‘yes’ or ‘worse’, suggesting that many of the problems were simply manifestations of old age. On a similar note, the participant added: ‘If you go looking for problems, you will find problems’, which may not necessarily be at the forefront of the service user’s concerns.

**Cognitive interview 3**

The third cognitive interview was performed on 5^th^ May 2021 with a nurse (participant code = CIN). CIN works as an advanced nurse practitioner in a large GP surgery. In CIN’s practice, medicine reviews are currently performed by advanced nurse practitioners and GPs due to pharmacist shortage. They use a structured medicine review template on the EMIS (Educational Management Information System) platform. This interview took 46 minutes.

Findings

Cognitive interview with CIN illuminated several potential issues in the supporting information section. The findings are summarised in table 00. CIN’s input led to development of a branching-logic version of the ADRe Profile, outlined in table 00. Other comments and thoughts pertaining to evaluation of the ADRe Profile, and its implementation are summarised below.

Other comments:

CIN hesitated and requested additional information on the following questions: Tongue abnormal movements or tremor, Abnormal movements at rest, Posture abnormal, Gait abnormal, CNS - Any convulsions (even if epileptic), Self-harm, Restlessness or pacing, Dizziness, Dry eyes, Short of breath/unable to finish sentences, Appetite/taste changes, Bowel control/diarrhoea, Sore throat and raised temperature, and Changes in smoking. Once supplementary information was shown, she was satisfied that she had sufficient information to proceed.

CIN thought that the items relating to cognitive and behaviour problems (Cognitive decline (memory or concentration problems), Behaviour problems) would require the service user to be accompanied by a family member or friend who knows them, as they may not reliably be able to answer the questions by themselves (they may not be aware of any problems or not want to admit them). CIN stated that in her practice, she would not know the service users sufficiently to notice any difference in cognitive abilities herself, her usual practice would be to contact a family member to obtain their view of the situation.

In the CIN’s experience, once the topic of behaviour problems is approached, the service user may start talking and answer several of the ADRe Profile items without further prompting. This idea led CIN to suggest that combining the behavioural problems questions would enhance the flow of the questionnaire. CIN also thought that especially if the service user was agitated or had problems, it would be easier for them to answer the grouped question, rather than individual ones. Similarly, agitation, panic attacks, restlessness, mood fluctuations may also be grouped together, as well as hallucinations/vivid dreams, insomnia, and sedation/excessive sleep. Other items that could be grouped were Dizziness and tinnitus/hearing problems; and Bowel control/diarrhoea + Constipation.

CIN stated that she found the ADRe Profile a potentially very useful and effective instrument in primary care, especially because clinicians do not always have time to enquire about the sort of problems included on the Profile. Having a template ready would enable the practitioner to utilise it with relevant service user group. CIP also made a positive comment about the versatility of the Profile, as it is suitable to be used by any health care professional working in the GP surgery. In the CIN’s opinion, large GP practices, such as hers do not often have the opportunity for team meetings and team discussions of individual patients or issues, and an instrument such as the ADRe Profile would help the flow of information and contribute to good working practices.

The CIN’s concern was the difficulty of vital asked while checking the vital signs. CIN believed the ADRe Profile would be most useful for service sign observations in the climate of Covid 19. In CIN’s practice, most consultations were performed over the phone, while the health professionals encouraged service users to purchase their own blood pressure checking equipment and keep the option of the service user’s visit to the GP practice only for cases where it is felt proper examination was needed. She then added that her practice still provided face to face hypertension reviews for service users who have not purchased their own blood pressure measuring device and as many of the attendees were over 65 on multiple medicines, the vital signs measurements could be taken as part of the clinic.

Another concern that CIN expressed was from her point of view as an advance nurse practitioner, she would seek to explore many of the questions on the ADRe Profile in more depth, and she wondered whether practice nurses or healthcare support workers completing the Profile would have enough knowledge to follow on any problems that were identified. CIN also estimated that the questionnaire without the vital signs would take about 10 minutes to implement, and that many of the questions could be users with cardiovascular and multi- system problems; interestingly, she did not think the instrument would be appropriate and accurate for mental health patients, especially service users with drug and alcohol misuse, majorly due to worry about not being able to elicit truthful answers from the service users. Current practice in the CIN’s GP surgery is to refer all service users with mental health issues to a specialist GP colleague.

CIN also reviewed the “How to” document, and thought it was clear and informative. She sought further clarification on when and how often the ADRe Profile is to be implemented, and expressed her view that discharge from hospital is a critical point when the ADRe Profile would be useful.

**Cognitive interview 4**

The fourth cognitive interview was performed on the 15th of May 2021 with a service user (participant code = CIS). CIS is retired, >65 years old, with multiple medicine prescriptions. The interview took 66 minutes.

Findings

Cognitive interview with CIS provided insight from a service user viewpoint. It illuminated several potential issues with the ADRe Profile items and the supporting information section. The findings are outlined in table 00, other comments and thoughts pertaining to evaluation of the ADRe Profile, and its implementation are summarised below.

Other comments:

Teaching is important, the service users need to be taught about the specific features of the electronic Profile, such as the information button and the availability of free text comments. The service user found the functionality and plain language explanations of terms and jargon that are already part of the ADRe Profile very useful. For example, the service user commented that he would not think of his problems on seeing the heading ‘Sweating’ but a probe of ‘night sweats’ in the supplementary information triggered his awareness.

Some of the items may be difficult for the service user to notice even if they themselves display the symptom, eg. abnormal posture, gait. These questions may not be ideal for service user self-completion.

Size of font in print or onscreen text may be an issue for some service users. The colour contrast could be a bit more pronounced on the computer screen.

**Cognitive interview 5**

Changes resulting from the previous 4 cognitive interviews and supervisory group discussions were made and the updated ADRe Profile and Supporting information was tested with another service user in cognitive interview 5.

The fifth cognitive interview was performed on the 12^th of^ September 2021 with a service user (participant code = CIS2). CIS2 is retired, >65 years old, with multiple medicine prescriptions. The interview took place in person at a mutually convenient time and date. The interview lasted 23 minutes.

Results

CIS2 found majority of questions easy to understand and answer, apart from the question about presence of non-verbal pain indicators, which he felt should be answered by health professionals, as lay people may not have the required knowledge. CIS2 hesitated over several other items but once he re-read them, he was satisfied that the question was clear. Overall, CIS2 thought that the questions were structured well and asked in logical order.
