# Supplementary material for: Reducing Adverse Drug Reactions for Older People in the Community: Evaluating the Validity and Reliability of the ADRe Profile
Source: J Nurs Manag. 2025 May 14;2025:9921349. doi: 10.1155/jonm/9921349 (PMC12094870; doi:10.1155/jonm/9921349)
Supplement: Supporting Information 3 — Content validity index by ADRe Profile sections. [file 9921349.f3.docx]

## Supplementary material 3: Content validity index by ADRe sections

Table 1: Content validity index by sectioning

| Adore section | Item | Agreement | I-CVI | Modified Kappa | Interpretation |
| --- | --- | --- | --- | --- | --- |
| Vital signs | Heart rate sitting | 11/14 | 0.78 | 0.79 | Excellent |
|  | Irregular rhythm | 11/14 | 0.78 | 0.79 | Excellent |
|  | BP sitting | 10/14 | 0.71 | 0.71 | For review |
|  | Change since last reading (BP sitting) | 13/14 | 0.93 | 0.93 | Excellent |
|  | BP standing | 11/14 | 0.79 | 0.79 | Excellent |
|  | Change since last reading (BP standing) | 13/14 | 0.93 | 0.93 | Excellent |
|  | Heart rate standing | 6/14 | 0.43 | 0.43 | For review |
|  | Body mass index (BMI) | 6/14 | 0.43 | 0.43 | For review |
|  | Weight | 5/14 | 0.36 | 0.36 | For review |
|  | Change in weight | 11/14 | 0.79 | 0.79 | Excellent |
|  | Girth | 4/14 | 0.28 | 0.28 | For review |
|  | Change in girth | 7/14 | 0.5 | 0.5 | For review |
|  | Temperature | 5/14 | 0.36 | 0.36 | For review |
|  | Oxygen saturation | 7/14 | 0.5 | 0.5 | For review |
|  | Change in oxygen saturation | 8/14 | 0.57 | 0.57 | For review |
|  | ECG available | 9/14 | 0.64 | 0.64 | For review |
|  | Lung function tests available | 7/14 | 0.5 | 0.5 | For review |
|  | Blood results** | 10/12 | 0.83 | 0.83 | Excellent |
| Observations | Hand tremor | 11/14 | 0.79 | 0.79 | Excellent |
|  | Tongue tremor | 9/14 | 0.64 | 0.64 | For review |
|  | Feet shuffling | 10/14 | 0.71 | 0.71 | For review |
|  | Abnormal movements at rest | 11/14 | 0.79 | 0.79 | Excellent |
|  | Posture abnormal | 6/14 | 0.43 | 0.43 | For review |
|  | Gait abnormal on walking | 9/14 | 0.64 | 0.64 | For review |
|  | Balance/coordination | 11/14 | 0.79 | 0.79 | Excellent |
|  | Cognitive decline | 12/14 | 0.86 | 0.86 | Excellent |
|  | Feeling the cold | 10/14 | 0.71 | 0.71 | For review |
|  | Any bleeding/bruising/nosebleeds | 12/14 | 0.86 | 0.86 | Excellent |
|  | Rash/itching | 13/14 | 0.93 | 0.93 | Excellent |
|  | Swelling/oedema | 12/14 | 0.86 | 0.86 | Excellent |
|  | Sweating | 10/14 | 0.71 | 0.71 | For review |
|  | Acne/hirsutism/herpes simplex | 8/14 | 0.57 | 0.57 | For review |
|  | Broken skin/poor healing | 8/14 | 0.57 | 0.57 | For review |
|  | Hair loss | 6/14 | 0.43 | 0.43 | For review |
|  | Other skin abnormalities (for example, colour) | 7/13 | 0.5 | 0.54 | For review |
|  | Injection site problems | 9/14 | 0.64 | 0.64 | For review |
| Reports, questions | Any convulsions | 13/14 | 0.93 | 0.93 | Excellent |
|  | Behavioural problems | 10/14 | 0.71 | 0.71 | For review |
|  | Self-harm | 9/14 | 0.93 | 0.64 | For review |
|  | Physical violence | 8/14 | 0.57 | 0.57 | For review |
|  | Aggression | 7/14 | 0.5 | 0.5 | For review |
|  | Agitation/anxiety/nervousness/hyperactivity | 12/14 | 0.86 | 0.86 | Excellent |
|  | Restlessness or pacing | 9/14 | 0.64 | 0.64 | For review |
|  | Panic attacks | 7/14 | 0.5 | 0.5 | For review |
|  | Confusion | 12/14 | 0.86 | 0.86 | Excellent |
|  | Mood fluctuations | 9/14 | 0.64 | 0.64 | For review |
|  | Low energy, weakness, apathy, fatigue | 9/14 | 0.64 | 0.64 | For review |
|  | Hallucinations/vivid dreams | 9/14 | 0.64 | 0.64 | For review |
| Reports, questions | Sleep problems/insomnia | 11/14 | 0.79 | 0.79 | Excellent |
|  | Sedation/excessive sleep | 9/14 | 0.64 | 0.64 | For review |
|  | Dizziness | 14/14 | 1 | 1 | Excellent |
|  | Falls | 14/14 | 1 | 1 | Excellent |
|  | Headache/migraine | 9/14 | 0.64 | 0.64 | For review |
|  | Any pain? | 9/14 | 0.64 | 0.64 | For review |
|  | Non-verbal pain indicators | 8/14 | 0.57 | 0.57 | For review |
|  | Tingling/pins and needles | 12/14 | 0.86 | 0.86 | For review |
|  | Tinnitus/hearing problems | 10/14 | 0.71 | 0.71 | For review |
|  | Vision/eyesight problems | 13/14 | 0.93 | 0.93 | Excellent |
|  | Dry eyes | 9/14 | 0.64 | 0.64 | For review |
|  | Urination problems/incontinence/UTI | 10/14 | 0.71 | 0.71 | For review |
|  | Urinary catheter | 7/14 | 0.5 | 0.5 | For review |
|  | Reproductive system | 6/14 | 0.43 | 0.43 | For review |
|  | Chest pain | 12/14 | 0.86 | 0.86 | Excellent |
|  | Short of breath | 12/14 | 0.86 | 0.86 | Excellent |
|  | High salt intake | 9/14 | 0.64 | 0.64 | For review |
|  | Problems with teeth/dentures | 8/14 | 0.57 | 0.57 | For review |
|  | Dry mouth | 10/14 | 0.71 | 0.71 | For review |
|  | Halitosis | 8/14 | 0.57 | 0.57 | For review |
|  | Hypersalivation | 9/14 | 0.64 | 0.64 | For review |
|  | Swallowing difficulties | 11/14 | 0.79 | 0.79 | Excellent |
|  | Indigestion/heartburn | 10/14 | 0.71 | 0.71 | For review |
|  | Nausea/vomiting | 11/14 | 0.79 | 0.79 | Excellent |
|  | Appetite/taste changes | 9/14 | 0.64 | 0.64 | For review |
|  | Bowel control/diarrhoea | 11/14 | 0.79 | 0.79 | Excellent |
|  | Constipation | 12/14 | 0.86 | 0.86 | Excellent |
|  | Sore throat/temperature | 9/14 | 0.64 | 0.64 | For review |
|  | Respiratory problems-wheeze, cough, shortness of breath (SOB)* | 11/13 | 0.85 | 0.85 | Excellent |
| Prevention and Health promotion | Optician in the last 12 months | 5/12 | 0.42 | 0.41 | For review |
|  | Dentist in the last 12 months | 5/12 | 0.42 | 0.41 | For review |
|  | Smoking | 10/12 | 0.83 | 0.83 | Excellent |
|  | Changes in smoking | 10/12 | 0.83 | 0.83 | Excellent |
|  | Dehydration risk | 10/12 | 0.83 | 0.83 | Excellent |
|  | Diet/snacking between meals | 5/12 | 0.41 | 0.41 | For review |
|  | Drinks sugar-free? | 1/12 | 0.08 | 0.08 | For review |
|  | Meals missed/left unfinished? | 8/12 | 0.67 | 0.67 | For review |
|  | 2+ meals eaten daily? | 4/12 | 0.33 | 0.33 | For review |
|  | Fruit/vegetables eaten every day? | 7/12 | 0.58 | 0.58 | For review |
|  | Fracture prevention: 1 pint of milk/day? | 6/12 | 0.5 | 0.5 | For review |
|  | Adequate vitamin D intake? | 7/12 | 0.58 | 0.58 | For review |
|  | Sunscreen available? | 5/12 | 0.41 | 0.41 | For review |
|  | Dark glasses available in the sun? | 2/12 | 0.17 | 0.15 | For review |
|  | Immunisations up-to-date? | 5/12 | 0.45 | 0.45 | For review |
| Medicines administration | Any tablets crushed or broken? | 9/12 | 0.75 | 0.75 | For review |
|  | Medicines taken as prescribed? | 10/12 | 0.83 | 0.83 | Excellent |
|  | More than 2 doses missed in the last 7 days? | 10/12 | 0.83 | 0.83 | Excellent |
|  | Medicines bought without prescription? | 11/12 | 0.92 | 0.92 | Excellent |
|  | Recreational drug use? | 11/12 | 0.92 | 0.92 | Excellent |
|  | Alcohol overuse? | 9/12 | 0.75 | 0.75 |  |
| Summary | Any other problems? | 10/12 | 0.83 | 0.83 | Excellent |
|  | Which is the most important problem for you? | 9/12 | 0.75 | 0.75 | For review |
|  | Do you have any other message for the prescriber? | 8/12 | 0.67 | 0.67 | For review |

Footnote: I-CVI – item-level content validity index, p_c_ – agreement by chance, calculated as pc = [ x 0.5^N^, where N = number of raters, A = number of raters who rated the item as 3 or 4.
