# Supplementary material for: Reducing Adverse Drug Reactions for Older People in the Community: Evaluating the Validity and Reliability of the ADRe Profile
Source: J Nurs Manag. 2025 May 14;2025:9921349. doi: 10.1155/jonm/9921349 (PMC12094870; doi:10.1155/jonm/9921349)
Supplement: Supporting Information 4 — Between-group differences in problems experienced. [file 9921349.f4.docx]

## Supplementary material 4: Between-group differences in problems experienced

Between-group differences in problems experienced (‘*Observations*’ and ‘*Reports*’ sections of the ADRe Profile)

|  | HV group n (%) (sample: 20) | 5+ group n (%) (sample: 48) |
| --- | --- | --- |
| Hand tremor | 0 | 8 (16.70) |
| Tongue tremor | 0 | 0 |
| Feet shuffling | 0 | 11 (22.90) |
| Abnormal movements at rest | 0 | 9 (18.80) |
| Posture abnormal | 0 | 11 (22.90) |
| Balance problems | 0 | 18 (37.50) |
| Cognitive decline | 0 | 19 (39.60) |
| Skin rash | 0 | 16 (33.30) |
| Acne/Herpes simplex | 0 | 6 (12.50) |
| Convulsions | 0 | 2 (4.20) |
| Behaviour problems | 0 | 1 (2.10) |
| Self-harm | 0 | 0 |
| Physical violence | 0 | 1 (2.10) |
| Aggression | 0 | 9 (18.80) |
| Agitation, anxiety | 0 | 17 (35.40) |
| Restlessness | 0 | 10 (20.80) |
| Confusion | 0 | 7 (14.60) |
| Mood fluctuations | 0 | 15 (31.30) |
| Dizziness | 0 | 18 (37.50) |
| Falls in the past month | 0 | 12 (25) |
| Dry eyes | 0 | 13 (27.10) |
| Chest pain | 0 | 11 (22.90) |
| Shortness of breath | 0 | 26 (54.20) |
| Halitosis | 0 | 5 (10.40) |
| Nausea, vomiting | 0 | 2 (4.20) |
| Appetite or taste changes | 0 | 4 (8.30) |
| Bowel problems, Diarrhoea | 0 | 14 (29.20) |
| Constipation | 0 | 5 (10.40) |
| Pain | 7 (35.00) | 37 (77.10) |
| Respiration problems | 6 (30.00) | 14 (29.17) |
| Excessive sweating | 5 (25.00) | 13 (27.10) |
| Sleep problems | 5 (25.00) | 15 (31.30) |
| Numbness or tingling in the extremities | 5 (25.00) | 18 (37.50) |
| Headaches | 4 (20.00) | 11 (22.92) |
| Urination problems | 4 (20.00) | 13 (27.08) |
| Hearing problems | 3 (15.00) | 23 (47.92) |
| Vision problems | 2 (10.00) | 33 (68.75) |
| Indigestion | 2 (10.00) | 20 (41.67) |
| Dry mouth | 1 (5.00) | 21 (43.75) |
