# Supplementary material for: Reducing Adverse Drug Reactions for Older People in the Community: Evaluating the Validity and Reliability of the ADRe Profile
Source: J Nurs Manag. 2025 May 14;2025:9921349. doi: 10.1155/jonm/9921349 (PMC12094870; doi:10.1155/jonm/9921349)
Supplement: Supporting Information 5 — Inter-rater reliability—kappa results per category. [file 9921349.f5.docx]

Supplementary material 5: Inter-rater reliability - kappa results per category

| Kappa | Kappa value interpretation (Landis & Koch, 1977] | Number of problems | Examples |
| --- | --- | --- | --- |
| 1 | Perfect agreement | 4 | Behaviour problems, Physical violence |
| >0.80 | Almost perfect agreement | 20 | Agitation/anxiety, Dry eyes |
| 0.61-0.80 | Substantial agreement | 33 | Dizziness, Tingling/pins and needles |
| 0.41-0.60 | Moderate agreement | 8 | Abnormal posture, Low energy |
| 0.21-0.40 | Fair agreement | 5 | Constipation, Breathing problems |
| 0.00-0.20 | Slight agreement | 0 |  |
| <0 | Poor agreement | 0 |  |
