# Supplementary material for: Reducing Adverse Drug Reactions for Older People in the Community: Evaluating the Validity and Reliability of the ADRe Profile
Source: J Nurs Manag. 2025 May 14;2025:9921349. doi: 10.1155/jonm/9921349 (PMC12094870; doi:10.1155/jonm/9921349)
Supplement: Supporting Information 6 — Full results for inter-rater reliability. [file 9921349.f6.docx]

## Supplementary material 6: Full results - reliability reporting of ADRe items, as rated by the service user (SU) and the researcher (R).

| **ADRe item** | **Cross-tabulation** | **Simple agreement measure** | **Cohen’s kappa** | **95% CI** | **Statistical significance (p-value)** |
| --- | --- | --- | --- | --- | --- |
| **Hand tremor** | \|  \| \| Researcher \| \| Total \| \| --- \| --- \| --- \| --- \| --- \| \| No, not present \| Yes, present \| \| Service user \| No, not present \| 32 \| 2 \| 34 \| \| Yes, present \| 2 \| 5 \| 7 \| \| Total \| \| 34 \| 7 \| 41 \| | 90% | 0.66 | 0.34-0.97 | <0.001 |
| **Tongue tremor** | \|  \| \| Researcher \| Total \| \| --- \| --- \| --- \| --- \| \| No, not present \| \| Service user \| No, not present \| 42 \| 42 \| \| Total \| \| 42 \| 42 \| | 100% | N/A | N/A | N/A |
| **Feet shuffling** | \|  \| \| Researcher \| \| Total \| \| --- \| --- \| --- \| --- \| --- \| \| No, not present \| Yes, present \| \| Service user \| No, not present \| 31 \| 2 \| 33 \| \| Yes, present \| 0 \| 8 \| 8 \| \| Total \| \| 31 \| 10 \| 41 \| | 95% | 0.86 | 0.67-1.00 | <0.001 |
| **Abnormal movements at rest** | \|  \| \| Researcher \| \| Total \| \| --- \| --- \| --- \| --- \| --- \| \| No, not present \| Yes, present \| \| Service user \| No, not present \| 31 \| 0 \| 31 \| \| Yes, present \| 1 \| 9 \| 10 \| \| Total \| \| 32 \| 9 \| 41 \| | 98% | 0.93 | 0.8-1.00 | <0.001 |
| **Abnormal posture** | \|  \| \| Researcher \| \| Total \| \| --- \| --- \| --- \| --- \| --- \| \| No, not present \| Yes, present \| \| Service user \| No, not present \| 29 \| 5 \| 34 \| \| Yes, present \| 2 \| 6 \| 8 \| \| Total \| \| 31 \| 11 \| 42 \| | 83% | 0.53 | 0.23-0.83 | <0.001 |
| **Gait abnormal** | \|  \| \| Researcher \| \| Total \| \| --- \| --- \| --- \| --- \| --- \| \| No, not present \| Yes, present \| \| Service user \| No, not present \| 26 \| 0 \| 26 \| \| Yes, present \| 3 \| 8 \| 11 \| \| Total \| \| 29 \| 8 \| 37 \| | 92% | 0.79 | 0.57-1 | <0.001 |
| **Balance** | \|  \| \| Researcher \| \| Total \| \| --- \| --- \| --- \| --- \| --- \| \| No, not present \| Yes, present \| \| Service user \| No, not present \| 24 \| 2 \| 26 \| \| Yes, present \| 2 \| 14 \| 16 \| \| Total \| \| 26 \| 16 \| 42 \| | 90% | 0.80 | 0.61-0.99 | <0.001 |
| **Cognitive Decline** | \|  \| \| Researcher \| \| Total \| \| --- \| --- \| --- \| --- \| --- \| \| No, not present \| Yes, present \| \| Service user \| No, not present \| 23 \| 4 \| 27 \| \| Yes, present \| 2 \| 13 \| 15 \| \| Total \| \| 25 \| 17 \| 42 \| | 86% | 0.70 | 0.48-0.92 | <0.001 |
| **Feeling cold** | \|  \| \| Researcher \| \| Total \| \| --- \| --- \| --- \| --- \| --- \| \| No, not present \| Yes, present \| \| Service user \| No, not present \| 25 \| 1 \| 26 \| \| Yes, present \| 2 \| 14 \| 16 \| \| Total \| \| 27 \| 15 \| 42 \| | 93% | 0.85 | 0.68-1 | <0.001 |
| **Bleeding, bruising** | \|  \| \| Researcher \| \| Total \| \| --- \| --- \| --- \| --- \| --- \| \| No, not present \| Yes, present \| \| Service user \| No, not present \| 24 \| 2 \| 26 \| \| Yes, present \| 1 \| 13 \| 14 \| \| Total \| \| 25 \| 15 \| 40 \| | 93% | 0.84 | 0.66-1 | <0.001 |
| **Skin rash** | \|  \| \| Researcher \| \| Total \| \| --- \| --- \| --- \| --- \| --- \| \| No, not present \| Yes, present \| \| Service user \| No, not present \| 24 \| 3 \| 27 \| \| Yes, present \| 3 \| 11 \| 14 \| \| Total \| \| 27 \| 14 \| 41 \| | 85% | 0.68 | 0.44-0.91 | <0.001 |
| **Oedema** | \|  \| \| Researcher \| \| Total \| \| --- \| --- \| --- \| --- \| --- \| \| No, not present \| Yes, present \| \| Service user \| No, not present \| 25 \| 4 \| 29 \| \| Yes, present \| 1 \| 12 \| 13 \| \| Total \| \| 26 \| 16 \| 42 \| | 88% | 0.74 | 0.53-0.95 | <0.001 |
| **Sweating** | \|  \| \| Researcher \| \| Total \| \| --- \| --- \| --- \| --- \| --- \| \| No, not present \| Yes, present \| \| Service user \| No, not present \| 30 \| 3 \| 33 \| \| Yes, present \| 1 \| 8 \| 9 \| \| Total \| \| 31 \| 11 \| 42 \| | 90% | 0.74 | 0.5-0.98 | <0.001 |
| **Acne, herpes simplex** | \|  \| \| Researcher \| \| Total \| \| --- \| --- \| --- \| --- \| --- \| \| No, not present \| Yes, present \| \| Service user \| No, not present \| 35 \| 0 \| 35 \| \| Yes, present \| 1 \| 5 \| 6 \| \| Total \| \| 36 \| 5 \| 41 \| | 98% | 0.90 | 0.69-1 | <0.001 |
| **Healing** | \|  \| \| Researcher \| \| Total \| \| --- \| --- \| --- \| --- \| --- \| \| No, not present \| Yes, present \| \| Service user \| No, not present \| 29 \| 3 \| 32 \| \| Yes, present \| 3 \| 7 \| 10 \| \| Total \| \| 32 \| 10 \| 42 \| | 86% | 0.61 | 0.32-0.89 | <0.001 |
| **Hair loss** | \|  \| \| Researcher \| \| Total \| \| --- \| --- \| --- \| --- \| --- \| \| No, not present \| Yes, present \| \| Service user \| No, not present \| 26 \| 1 \| 27 \| \| Yes, present \| 3 \| 12 \| 15 \| \| Total \| \| 29 \| 13 \| 42 \| | 90% | 0.79 | 0.59-0.98 | <0.001 |
| **Skin abnormal** | \|  \| \| Researcher \| \| Total \| \| --- \| --- \| --- \| --- \| --- \| \| No, not present \| Yes, present \| \| Service user \| No, not present \| 26 \| 7 \| 33 \| \| Yes, present \| 4 \| 5 \| 9 \| \| Total \| \| 30 \| 12 \| 42 \| | 74% | 0.31 | 0.01-0.62 | <0.043 |
| **Injection site pain** | \|  \| \| Researcher \| \| Total \| \| --- \| --- \| --- \| --- \| --- \| \| No, not present \| Yes, present \| \| Service user \| No, not present \| 37 \| 0 \| 37 \| \| Yes, present \| 2 \| 1 \| 3 \| \| Total \| \| 39 \| 1 \| 40 \| | 95% | 0.48 | 0.12-1 | <0.001 |
| **Convulsion** | \|  \| \| Researcher \| \| Total \| \| --- \| --- \| --- \| --- \| --- \| \| No, not present \| Yes, present \| \| Service user \| No, not present \| 40 \| 1 \| 41 \| \| Yes, present \| 0 \| 1 \| 1 \| \| Total \| \| 40 \| 2 \| 42 \| | 98% | 0.66 | 0.3-1 | <0.001 |
| **Behaviour problems** | \|  \| \| Researcher \| \| Total \| \| --- \| --- \| --- \| --- \| --- \| \| No, not present \| Yes, present \| \| Service user \| No, not present \| 39 \| 0 \| 39 \| \| Yes, present \| 0 \| 1 \| 1 \| \| Total \| \| 39 \| 1 \| 40 \| | 100% | 1 | 1 | <0.01 |
| **Self-harm** | \|  \| \| Researcher \| Total \| \| --- \| --- \| --- \| --- \| \| No, not present \| \| Service user \| No, not present \| 42 \| 42 \| \| Total \| \| 42 \| 42 \| | 100% | N/A | N/A | N/A |
| **Physical violence** | \|  \| \| Researcher \| \| Total \| \| --- \| --- \| --- \| --- \| --- \| \| No, not present \| Yes, present \| \| Service user \| No, not present \| 41 \| 0 \| 41 \| \| Yes, present \| 0 \| 1 \| 1 \| \| Total \| \| 41 \| 1 \| 42 \| | 100% | 1 | 1 | <0.001 |
| **Aggression** | \|  \| \| Researcher \| \| Total \| \| --- \| --- \| --- \| --- \| --- \| \| No, not present \| Yes, present \| \| Service user \| No, not present \| 32 \| 1 \| 33 \| \| Yes, present \| 2 \| 7 \| 9 \| \| Total \| \| 34 \| 8 \| 42 \| | 93% | 0.78 | 0.54-1 | <0.001 |
| **Agitation, anxiety** | \|  \| \| Researcher \| \| Total \| \| --- \| --- \| --- \| --- \| --- \| \| No, not present \| Yes, present \| \| Service user \| No, not present \| 24 \| 1 \| 25 \| \| Yes, present \| 2 \| 15 \| 17 \| \| Total \| \| 26 \| 16 \| 42 \| | 93% | 0.85 | 0.69-1 | <0.001 |
| **Restlessness** | \|  \| \| Researcher \| \| Total \| \| --- \| --- \| --- \| --- \| --- \| \| No, not present \| Yes, present \| \| Service user \| No, not present \| 30 \| 3 \| 33 \| \| Yes, present \| 2 \| 7 \| 9 \| \| Total \| \| 32 \| 10 \| 42 \| | 88% | 0.66 | 0.39-0.93 | <0.001 |
| **Panic attacks** | \|  \| \| Researcher \| \| Total \| \| --- \| --- \| --- \| --- \| --- \| \| No, not present \| Yes, present \| \| Service user \| No, not present \| 33 \| 0 \| 33 \| \| Yes, present \| 2 \| 7 \| 9 \| \| Total \| \| 35 \| 7 \| 42 \| | 95% | 0.85 | 0.64-1 | 0.001 |
| **Confusion** | \|  \| \| Researcher \| \| Total \| \| --- \| --- \| --- \| --- \| --- \| \| No, not present \| Yes, present \| \| Service user \| No, not present \| 32 \| 0 \| 32 \| \| Yes, present \| 2 \| 7 \| 9 \| \| Total \| \| 34 \| 7 \| 41 \| | 95% | 0.85 | 0.64-1 | <0.001 |
| **Mood fluctuations** | \|  \| \| Researcher \| \| Total \| \| --- \| --- \| --- \| --- \| --- \| \| No, not present \| Yes, present \| \| Service user \| No, not present \| 26 \| 2 \| 28 \| \| Yes, present \| 1 \| 12 \| 13 \| \| Total \| \| 27 \| 14 \| 41 \| | 93% | 0.83 | 0.67-1 | <0.001 |
| **Low energy, fatique** | \|  \| \| Researcher \| \| Total \| \| --- \| --- \| --- \| --- \| --- \| \| No, not present \| Yes, present \| \| Service user \| No, not present \| 13 \| 2 \| 15 \| \| Yes, present \| 7 \| 16 \| 23 \| \| Total \| \| 20 \| 18 \| 38 \| | 76% | 0.53 | 0.27-0.79 | <0.001 |
| **Hallucinations, vivid dreams** | \|  \| \| Researcher \| \| Total \| \| --- \| --- \| --- \| --- \| --- \| \| No, not present \| Yes, present \| \| Service user \| No, not present \| 29 \| 2 \| 31 \| \| Yes, present \| 0 \| 11 \| 11 \| \| Total \| \| 29 \| 13 \| 42 \| | 95% | 0.88 | 0.73-1 | <0.001 |
| **Sleep problems** | \|  \| \| Researcher \| \| Total \| \| --- \| --- \| --- \| --- \| --- \| \| No, not present \| Yes, present \| \| Service user \| No, not present \| 23 \| 2 \| 25 \| \| Yes, present \| 4 \| 12 \| 16 \| \| Total \| \| 27 \| 14 \| 41 \| | 85% | 0.69 | 0.45-0.92 | <0.001 |
| **Sedation** | \|  \| \| Researcher \| \| Total \| \| --- \| --- \| --- \| --- \| --- \| \| No, not present \| Yes, present \| \| Service user \| No, not present \| 37 \| 1 \| 38 \| \| Yes, present \| 0 \| 1 \| 1 \| \| Total \| \| 37 \| 2 \| 39 \| | 97% | 0.66 | 0.03-1 | <0.001 |
| **Dizziness** | \|  \| \| Researcher \| \| Total \| \| --- \| --- \| --- \| --- \| --- \| \| No, not present \| Yes, present \| \| Service user \| No, not present \| 24 \| 4 \| 28 \| \| Yes, present \| 2 \| 12 \| 14 \| \| Total \| \| 26 \| 16 \| 42 \| | 86% | 0.69 | 0.46-0.92 | <0.001 |
| **Falls** | \|  \| \| Researcher \| \| Total \| \| --- \| --- \| --- \| --- \| --- \| \| No, not present \| Yes, present \| \| Service user \| No, not present \| 30 \| 1 \| 31 \| \| Yes, present \| 0 \| 10 \| 10 \| \| Total \| \| 30 \| 11 \| 41 \| | 98% | 0.94 | 0.81-1 | <0.001 |
| **Headache** | \|  \| \| Researcher \| \| Total \| \| --- \| --- \| --- \| --- \| --- \| \| No, not present \| Yes, present \| \| Service user \| No, not present \| 31 \| 1 \| 32 \| \| Yes, present \| 1 \| 9 \| 10 \| \| Total \| \| 32 \| 10 \| 42 \| | 95% | 0.87 | 0.69-1 | <0.001 |
| **Pain** | \|  \| \| Researcher \| \| Total \| \| --- \| --- \| --- \| --- \| --- \| \| No, not present \| Yes, present \| \| Service user \| No, not present \| 5 \| 3 \| 8 \| \| Yes, present \| 5 \| 29 \| 34 \| \| Total \| \| 10 \| 32 \| 42 \| | 81% | 0.44 | 0.11-0.76 | <0.004 |
| **Non-verbal pain indicators** | \|  \| \| Researcher \| Total \| \| --- \| --- \| --- \| --- \| \| No, not present \| \| Service user \| No, not present \| 16 \| 16 \| \| Total \| \| 16 \| 16 \| | 100% | N/A | N/A | N/A |
| **Tingling, pins & needles** | \|  \| \| Researcher \| \| Total \| \| --- \| --- \| --- \| --- \| --- \| \| No, not present \| Yes, present \| \| Service user \| No, not present \| 22 \| 2 \| 24 \| \| Yes, present \| 4 \| 14 \| 18 \| \| Total \| \| 26 \| 16 \| 42 \| | 86% | 0.70 | 0.47-0.92 | <0.001 |
| **Hearing problems** | \|  \| \| Researcher \| \| Total \| \| --- \| --- \| --- \| --- \| --- \| \| No, not present \| Yes, present \| \| Service user \| No, not present \| 21 \| 2 \| 23 \| \| Yes, present \| 1 \| 18 \| 19 \| \| Total \| \| 22 \| 20 \| 42 \| | 93% | 0.86 | 0.7-1 | <0.001 |
| **Vision problems** | \|  \| \| Researcher \| \| Total \| \| --- \| --- \| --- \| --- \| --- \| \| No, not present \| Yes, present \| \| Service user \| No, not present \| 12 \| 10 \| 22 \| \| Yes, present \| 2 \| 16 \| 18 \| \| Total \| \| 14 \| 26 \| 40 \| | 95% | 0.42 | 0.16-0.67 | <0.004 |
| **Dry eyes** | \|  \| \| Researcher \| \| Total \| \| --- \| --- \| --- \| --- \| --- \| \| No, not present \| Yes, present \| \| Service user \| No, not present \| 28 \| 1 \| 29 \| \| Yes, present \| 2 \| 10 \| 12 \| \| Total \| \| 30 \| 11 \| 41 \| | 93% | 0.82 | 0.62-1 | <0.001 |
| **Urination problems** | \|  \| \| Researcher \| \| Total \| \| --- \| --- \| --- \| --- \| --- \| \| No, not present \| Yes, present \| \| Service user \| No, not present \| 28 \| 2 \| 30 \| \| Yes, present \| 0 \| 10 \| 10 \| \| Total \| \| 28 \| 12 \| 40 \| | 95% | 0.88 | 0.71-1 | <0.001 |
| **Catheter in situ** | \|  \| \| Researcher \| Total \| \| --- \| --- \| --- \| --- \| \| No, not present \| \| Service user \| No, not present \| 41 \| 41 \| \| Total \| \| 41 \| 41 \| | 100% | N/A | N/A | N/A |
| **Reproductive system problems** | \|  \| \| Researcher \| \| Total \| \| --- \| --- \| --- \| --- \| --- \| \| No, not present \| Yes, present \| \| Service user \| No, not present \| 22 \| 0 \| 22 \| \| Yes, present \| 1 \| 3 \| 4 \| \| Total \| \| 23 \| 3 \| 26 \| | 96% | 0.84 | 0.52-1 | <0.001 |
| **Chest pain** | \|  \| \| Researcher \| \| Total \| \| --- \| --- \| --- \| --- \| --- \| \| No, not present \| Yes, present \| \| Service user \| No, not present \| 29 \| 2 \| 31 \| \| Yes, present \| 1 \| 9 \| 10 \| \| Total \| \| 30 \| 11 \| 41 \| | 93% | 0.81 | 0.6-1 | <0.001 |
| **Dyspnea** | \|  \| \| Researcher \| \| Total \| \| --- \| --- \| --- \| --- \| --- \| \| No, not present \| Yes, present \| \| Service user \| No, not present \| 18 \| 8 \| 26 \| \| Yes, present \| 1 \| 15 \| 16 \| \| Total \| \| 19 \| 23 \| 42 \| | 79% | 0.58 | 0.35-0.81 | <0.001 |
| **High salt intake** | \|  \| \| Researcher \| \| Total \| \| --- \| --- \| --- \| --- \| --- \| \| No, not present \| Yes, present \| \| Service user \| No, not present \| 36 \| 1 \| 37 \| \| Yes, present \| 0 \| 1 \| 1 \| \| Total \| \| 36 \| 2 \| 38 \| | 97% | 0.66 | 0.03-1 | <0.001 |
| **Dental problems** | \|  \| \| Researcher \| \| Total \| \| --- \| --- \| --- \| --- \| --- \| \| No, not present \| Yes, present \| \| Service user \| No, not present \| 28 \| 1 \| 29 \| \| Yes, present \| 3 \| 7 \| 10 \| \| Total \| \| 31 \| 8 \| 39 \| | 90% | 0.71 | 0.45-0.97 | <0.001 |
| **Dry mouth** | \|  \| \| Researcher \| \| Total \| \| --- \| --- \| --- \| --- \| --- \| \| No, not present \| Yes, present \| \| Service user \| No, not present \| 20 \| 3 \| 23 \| \| Yes, present \| 2 \| 17 \| 19 \| \| Total \| \| 22 \| 20 \| 42 \| | 88% | 0.76 | 0.57-0.96 | <0.001 |
| **Halitosis** | \|  \| \| Researcher \| \| Total \| \| --- \| --- \| --- \| --- \| --- \| \| No, not present \| Yes, present \| \| Service user \| No, not present \| 36 \| 2 \| 38 \| \| Yes, present \| 0 \| 3 \| 3 \| \| Total \| \| 36 \| 5 \| 41 \| | 95% | 0.73 | 0.37-1 | <0.001 |
| **Hyper-salivation** | \|  \| \| Researcher \| \| Total \| \| --- \| --- \| --- \| --- \| --- \| \| No, not present \| Yes, present \| \| Service user \| No, not present \| 36 \| 0 \| 36 \| \| Yes, present \| 2 \| 4 \| 6 \| \| Total \| \| 38 \| 4 \| 42 \| | 95% | 0.77 | 0.48-1 | <0.001 |
| **Swallowing diff** | \|  \| \| Researcher \| \| Total \| \| --- \| --- \| --- \| --- \| --- \| \| No, not present \| Yes, present \| \| Service user \| No, not present \| 31 \| 2 \| 33 \| \| Yes, present \| 3 \| 6 \| 9 \| \| Total \| \| 34 \| 8 \| 42 \| | 88% | 0.63 | 0.34-0.93 | <0.001 |
| **Indigestion, heartburn** | \|  \| \| Researcher \| \| Total \| \| --- \| --- \| --- \| --- \| --- \| \| No, not present \| Yes, present \| \| Service user \| No, not present \| 21 \| 5 \| 26 \| \| Yes, present \| 3 \| 13 \| 16 \| \| Total \| \| 24 \| 18 \| 42 \| | 81% | 0.61 | 0.36-0.85 | <0.001 |
| **Nausea, vomiting** | \|  \| \| Researcher \| \| Total \| \| --- \| --- \| --- \| --- \| --- \| \| No, not present \| Yes, present \| \| Service user \| No, not present \| 39 \| 0 \| 39 \| \| Yes, present \| 1 \| 2 \| 3 \| \| Total \| \| 40 \| 2 \| 42 \| | 98% | 0.79 | 0.39-1 | <0.001 |
| **Appetite/taste changes** | \|  \| \| Researcher \| \| Total \| \| --- \| --- \| --- \| --- \| --- \| \| No, not present \| Yes, present \| \| Service user \| No, not present \| 36 \| 0 \| 36 \| \| Yes, present \| 3 \| 3 \| 6 \| \| Total \| \| 39 \| 3 \| 42 \| | 93% | 0.63 | 0.26-1 | <0.001 |
| **Bowel problems, diarrhoea** | \|  \| \| Researcher \| \| Total \| \| --- \| --- \| --- \| --- \| --- \| \| No, not present \| Yes, present \| \| Service user \| No, not present \| 26 \| 3 \| 29 \| \| Yes, present \| 3 \| 8 \| 11 \| \| Total \| \| 29 \| 11 \| 40 \| | 85% | 0.62 | 0.35-0.9 | <0.001 |
| **Constipation** | \|  \| \| Researcher \| \| Total \| \| --- \| --- \| --- \| --- \| --- \| \| No, not present \| Yes, present \| \| Service user \| No, not present \| 37 \| 3 \| 40 \| \| Yes, present \| 0 \| 1 \| 1 \| \| Total \| \| 37 \| 4 \| 41 \| | 93% | 0.38 | -0.16-0.9 | <0.002 |
| **Sore throat** | \|  \| \| Researcher \| \| Total \| \| --- \| --- \| --- \| --- \| --- \| \| No, not present \| Yes, present \| \| Service user \| No, not present \| 39 \| 0 \| 39 \| \| Yes, present \| 0 \| 3 \| 3 \| \| Total \| \| 39 \| 3 \| 42 \| | 100% | 1 | 1 | <0.001 |
| **Respiration problems** | \|  \| \| Researcher \| \| Total \| \| --- \| --- \| --- \| --- \| --- \| \| No, not present \| Yes, present \| \| Service user \| No, not present \| 18 \| 2 \| 20 \| \| Yes, present \| 11 \| 11 \| 22 \| \| Total \| \| 29 \| 13 \| 42 \| | 69% | 0.39 | 0.14-0.64 | <0.005 |
| **Opticians visit in the last 12m** | \|  \| \| Researcher \| \| Total \| \| --- \| --- \| --- \| --- \| --- \| \| No, not present \| Yes, present \| \| Service user \| No, not present \| 16 \| 1 \| 17 \| \| Yes, present \| 2 \| 22 \| 24 \| \| Total \| \| 18 \| 23 \| 41 \| | 93% | 0.85 | 0.69-1 | <0.001 |
| **Dentists visit in the last 12m** | \|  \| \| Researcher \| \| Total \| \| --- \| --- \| --- \| --- \| --- \| \| No, not present \| Yes, present \| \| Service user \| No, not present \| 24 \| 0 \| 24 \| \| Yes, present \| 1 \| 16 \| 17 \| \| Total \| \| 25 \| 16 \| 41 \| | 98% | 0.95 | 0.85-1 | <0.001 |
| **Smoking** | \|  \| \| Researcher \| \| Total \| \| --- \| --- \| --- \| --- \| --- \| \| No, not present \| Yes, present \| \| Service user \| No, not present \| 38 \| 0 \| 38 \| \| Yes, present \| 0 \| 3 \| 3 \| \| Total \| \| 38 \| 3 \| 41 \| | 100% | 1 | 1 | <0.001 |
| **Drinking 6-8 cups per day** | \|  \| \| Researcher \| \| Total \| \| --- \| --- \| --- \| --- \| --- \| \| No, not present \| Yes, present \| \| Service user \| No, not present \| 7 \| 3 \| 10 \| \| Yes, present \| 1 \| 30 \| 31 \| \| Total \| \| 8 \| 33 \| 41 \| | 90% | 0.72 | 0.46-0.98 | <0.001 |
| **Snacking** | \|  \| \| Researcher \| \| Total \| \| --- \| --- \| --- \| --- \| --- \| \| No, not present \| Yes, present \| \| Service user \| No, not present \| 18 \| 4 \| 22 \| \| Yes, present \| 2 \| 17 \| 19 \| \| Total \| \| 20 \| 21 \| 41 \| | 83% | 0.71 | 0.49-0.92 | <0.001 |
| **Intake missed** | \|  \| \| Researcher \| \| Total \| \| --- \| --- \| --- \| --- \| --- \| \| No, not present \| Yes, present \| \| Service user \| No, not present \| 31 \| 6 \| 37 \| \| Yes, present \| 1 \| 3 \| 4 \| \| Total \| \| 32 \| 9 \| 41 \| | 83% | 0.38 | 0.03-0.73 | <0.007 |
| **Meals eaten** | \|  \| \| Researcher \| \| Total \| \| --- \| --- \| --- \| --- \| --- \| \| No, not present \| Yes, present \| \| Service user \| No, not present \| 2 \| 3 \| 5 \| \| Yes, present \| 3 \| 34 \| 37 \| \| Total \| \| 5 \| 37 \| 42 \| | 86% | 0.32 | -0.1-0.74 | <0.039 |
| **Fruit + vegetable intake** | \|  \| \| Researcher \| \| Total \| \| --- \| --- \| --- \| --- \| --- \| \| No, not present \| Yes, present \| \| Service user \| No, not present \| 3 \| 2 \| 5 \| \| Yes, present \| 1 \| 36 \| 37 \| \| Total \| \| 4 \| 38 \| 42 \| | 93% | 0.63 | 0.24-1 | <0.001 |
| **Milk intake** | \|  \| \| Researcher \| \| Total \| \| --- \| --- \| --- \| --- \| --- \| \| No, not present \| Yes, present \| \| Service user \| No, not present \| 15 \| 8 \| 23 \| \| Yes, present \| 2 \| 16 \| 18 \| \| Total \| \| 17 \| 24 \| 41 \| | 76% | 0.52 | 0.28-0.77 | <0.001 |
| **Vitamin D intake** | \|  \| \| Researcher \| \| Total \| \| --- \| --- \| --- \| --- \| --- \| \| No, not present \| Yes, present \| \| Service user \| No, not present \| 9 \| 1 \| 10 \| \| Yes, present \| 0 \| 31 \| 31 \| \| Total \| \| 9 \| 32 \| 41 \| | 98% | 0.93 | 0.8-1 | <0.001 |
| **Skin protection 4 star** | \|  \| \| Researcher \| \| Total \| \| --- \| --- \| --- \| --- \| --- \| \| No, not present \| Yes, present \| \| Service user \| No, not present \| 7 \| 2 \| 9 \| \| Yes, present \| 3 \| 24 \| 27 \| \| Total \| \| 10 \| 26 \| 36 \| | 86% | 0.64 | 0.36-0.93 | <0.001 |
| **Sunglasses** | \|  \| \| Researcher \| \| Total \| \| --- \| --- \| --- \| --- \| --- \| \| No, not present \| Yes, present \| \| Service user \| No, not present \| 3 \| 2 \| 5 \| \| Yes, present \| 1 \| 31 \| 32 \| \| Total \| \| 4 \| 33 \| 37 \| | 92% | 0.62 | 0.23-1 | <0.001 |
| **Immunisations** | \|  \| \| Researcher \| \| Total \| \| --- \| --- \| --- \| --- \| --- \| \| No, not present \| Yes, present \| \| Service user \| Yes, present \| 2 \| 38 \| 40 \| \| Total \| \| 2 \| 38 \| 40 \| | 95% | N/A | N/A | N/A |
| **Tablets crushed** | \|  \| \| Researcher \| \| Total \| \| --- \| --- \| --- \| --- \| --- \| \| No, not present \| Yes, present \| \| Service user \| No, not present \| 40 \| 1 \| 41 \| \| Yes, present \| 0 \| 1 \| 1 \| \| Total \| \| 40 \| 2 \| 42 \| | 98% | 0.66 | 0.03-1 | <0.001 |
| **Medicines as prescribed** | \|  \| \| Researcher \| Total \| \| --- \| --- \| --- \| --- \| \| Yes, present \| \| Service user \| Yes, present \| 40 \| 40 \| \| Total \| \| 40 \| 40 \| \|  \| \|  \|  \| | 100% | N/A | N/A | N/A |
| **Medicines missed** | \|  \| \| Researcher \| \| Total \| \| --- \| --- \| --- \| --- \| --- \| \| No, not present \| Yes, present \| \| Service user \| No, not present \| 40 \| 2 \| 42 \| \| Total \| \| 40 \| 2 \| 42 \| | 95% | N/A | N/A | N/A |
| **Over the counter medicines** | \|  \| \| Researcher \| \| Total \| \| --- \| --- \| --- \| --- \| --- \| \| No, not present \| Yes, present \| \| Service user \| No, not present \| 22 \| 6 \| 28 \| \| Yes, present \| 2 \| 12 \| 14 \| \| Total \| \| 24 \| 18 \| 42 \| | 81% | 0.60 | 0.36-0.84 | <0.001 |
| **Illicit drug use** | \|  \| \| Researcher \| Total \| \| --- \| --- \| --- \| --- \| \| No, not present \| \| Service user \| No, not present \| 42 \| 42 \| \| Total \| \| 42 \| 42 \| | 100% | N/A | N/A | N/A |
| **Alcohol use** | \|  \| \| Researcher \| \| Total \| \| --- \| --- \| --- \| --- \| --- \| \| No, not present \| Yes, present \| \| Service user \| No, not present \| 34 \| 0 \| 34 \| \| Yes, present \| 4 \| 3 \| 7 \| \| Total \| \| 38 \| 3 \| 41 \| | 90% | 0.55 | 0.18-0.92 | <0.001 |
